# Coinfection of Chickens with H9N2 and H7N9 Avian Influenza Viruses Leads to Emergence of Reassortant H9N9 Virus with Increased Fitness for Poultry and a Zoonotic Potential

**DOI:** 10.1128/jvi.01856-21

**Published:** 2022-03-09

**Authors:** Sushant Bhat, Joe James, Jean-Remy Sadeyen, Sahar Mahmood, Holly J. Everest, Pengxiang Chang, Sarah K. Walsh, Alexander M. P. Byrne, Benjamin Mollett, Fabian Lean, Joshua E. Sealy, Holly Shelton, Marek J. Slomka, Sharon M. Brookes, Munir Iqbal

**Affiliations:** a Avian Influenza Group, The Pirbright Institute, Woking, United Kingdom; b Virology Department, Animal & Plant Health Agency, Weybridge, United Kingdom; The Peter Doherty Institute for Infection and Immunity

**Keywords:** avian influenza viruses, H7N9, H9N2, H9N9, coinfection, ferrets, genetic reassortment, poultry, virus transmission, zoonosis

## Abstract

An H7N9 low-pathogenicity avian influenza virus (LPAIV) emerged in 2013 through genetic reassortment between H9N2 and other LPAIVs circulating in birds in China. This virus causes inapparent clinical disease in chickens, but zoonotic transmission results in severe and fatal disease in humans. To examine a natural reassortment scenario between H7N9 and G1 lineage H9N2 viruses predominant in the Indian subcontinent, we performed an experimental coinfection of chickens with A/Anhui/1/2013/H7N9 (Anhui/13) virus and A/Chicken/Pakistan/UDL-01/2008/H9N2 (UDL/08) virus. Plaque purification and genotyping of the reassortant viruses shed via the oropharynx of contact chickens showed H9N2 and H9N9 as predominant subtypes. The reassortant viruses shed by contact chickens also showed selective enrichment of polymerase genes from H9N2 virus. The viable “6+2” reassortant H9N9 (having nucleoprotein [NP] and neuraminidase [NA] from H7N9 and the remaining genes from H9N2) was successfully shed from the oropharynx of contact chickens, plus it showed an increased replication rate in human A549 cells and a significantly higher receptor binding to α2,6 and α2,3 sialoglycans compared to H9N2. The reassortant H9N9 virus also had a lower fusion pH, replicated in directly infected ferrets at similar levels compared to H7N9 and transmitted via direct contact. Ferrets exposed to H9N9 via aerosol contact were also found to be seropositive, compared to H7N9 aerosol contact ferrets. To the best of our knowledge, this is the first study demonstrating that cocirculation of H7N9 and G1 lineage H9N2 viruses could represent a threat for the generation of novel reassortant H9N9 viruses with greater virulence in poultry and a zoonotic potential.

**IMPORTANCE** We evaluated the consequences of reassortment between the H7N9 and the contemporary H9N2 viruses of the G1 lineage that are enzootic in poultry across the Indian subcontinent and the Middle East. Coinfection of chickens with these viruses resulted in the emergence of novel reassortant H9N9 viruses with genes derived from both H9N2 and H7N9 viruses. The “6+2” reassortant H9N9 (having NP and NA from H7N9) virus was shed from contact chickens in a significantly higher proportion compared to most of the reassortant viruses, showed significantly increased replication fitness in human A549 cells, receptor binding toward human (α2,6) and avian (α2,3) sialic acid receptor analogues, and the potential to transmit via contact among ferrets. This study demonstrated the ability of viruses that already exist in nature to exchange genetic material, highlighting the potential emergence of viruses from these subtypes with zoonotic potential.

## INTRODUCTION

Novel human influenza A virus (IAV) infections during the past decade have included the H7N9 subtype, first isolated from humans in China in early 2013 (1). The virus was shown to be of avian origin, with zoonotic cases shown to be associated with exposure to infected birds at live poultry markets. Phylogenetic analyses revealed that the genotype of this avian influenza virus (AIV) arose naturally through complex genetic reassortment events. The hemagglutinin (HA) gene was related to those identified in Eurasian-lineage H7N3 viruses found in ducks in Zhejiang, and its neuraminidase (NA) gene was closely related to that possessed by H7N9 viruses circulating in wild migratory birds in Korea (2). Furthermore, the six internal gene segments were most closely related to H9N2 viruses, which are enzootic in chickens in China (2). This novel H7N9 virus was characterized as a low-pathogenicity (LP) AIV (3) and caused mild or unapparent clinical disease in domestic chickens and ducks (4), although a more severe pathogenesis may occur in turkeys (5). Since 2013, transmission of this H7N9 virus from infected birds to humans has resulted in over 1,500 infections in China with a case fatality rate of over 39% (6). Despite occasional reports of suspected nosocomial transmission (7), there has been no sustained spread of H7N9 between humans (8). The continued enzootic circulation of H7N9 in poultry in China also resulted in the eventual acquisition of polybasic amino acids at the cleavage site of the HA glycoprotein, a genetic hallmark of highly pathogenic (HP) AIVs (9–11). The H7N9 HPAIV variants had an ability to cause up to 100% mortality in chickens (12) and have resulted in at least 32 recorded human infections (6, 13).

The evolutionary trend of H7N9 viruses and other zoonotic reassortant influenza viruses in nature inferred an increased reassortment propensity of H9N2 compared to other cocirculating AIV subtypes (2, 14–16). This observation suggested that cocirculation of these viruses may predispose toward reassortment events to produce further genotypes with unknown disease risks to both poultry and humans. The continued circulation of H7N9 and H9N2 viruses with other AIV subtypes, enzootic in farmed and wild bird populations, has resulted in emergence of novel reassortant viruses with variable pathogenesis, along with the potential for mammalian adaptation and zoonotic transmission (17–23).

Eurasian H9N2 AIVs have diversified into three main lineages (G1, BJ94, and Y438), which have themselves evolved to be characteristic of the geographical region that they occupy (24, 25). Thus, H9N2 AIVs are enzootic in poultry in Asia, the Middle East, and Africa (26, 27), where they cause mild to severe morbidity and mortality in different avian species depending on the virus genotype (26, 28–31). Like the emergence of novel genotype H7N9 LPAIVs, the G1 lineage H9N2 AIVs currently circulating in the Indian subcontinent and the Middle East also include viruses which have undergone genetic reassortment with the internal gene segments from regional H7N3 HPAIVs (32–39). These reassortant H9N2 viruses have an increased zoonotic potential (39) and have been reported to be more virulent and transmissible between poultry and wild birds compared with their progenitors (40, 41).

Risk assessments considered that potential spread of H7N9 from China to poultry in neighboring countries represented a credible threat (42) with potential consequences including reassortment events with endemic H9N2 strains. To examine the potential natural reassortment scenario between H7N9 and G1 lineage H9N2 viruses, we performed experimental coinfection of chickens with A/Anhui/1/2013 (H7N9) (Anhui/13) virus and A/Chicken/Pakistan/UDL-01/2008 (H9N2) (UDL/08) virus. The genetic composition and phenotypic characteristics of the emergent reassortant viruses were analyzed. An H9N9 reassortant was appropriately selected for infection using a ferret model to further ascertain any potential zoonotic characteristics.

## RESULTS

### H9 subtype viruses display enhanced viral shedding compared to the H7 subtype in coinfected chickens.

To investigate the propensity for *in vivo* reassortment to occur between H9N2 (UDL/08) and H7N9 (Anhui/13) AIVs in chickens, we needed to be able to identify the proportions of progeny viruses which contained the H7 or H9 HA gene segments. We developed an array of reverse transcriptase quantitative PCR (RT-qPCR) assays for each influenza gene segment, which could specifically detect and discriminate the origin of the gene segment as either H9N2 (UDL/08) or H7N9 (Anhui/13) (see Table S1 in the supplemental material). Each gene segment-specific assay yielded equivalent sensitivity and performance (Fig. S1) and was equivalent to the generic “pan-avian influenza” M-gene RT-qPCR (data not shown).

**FIG 1:**
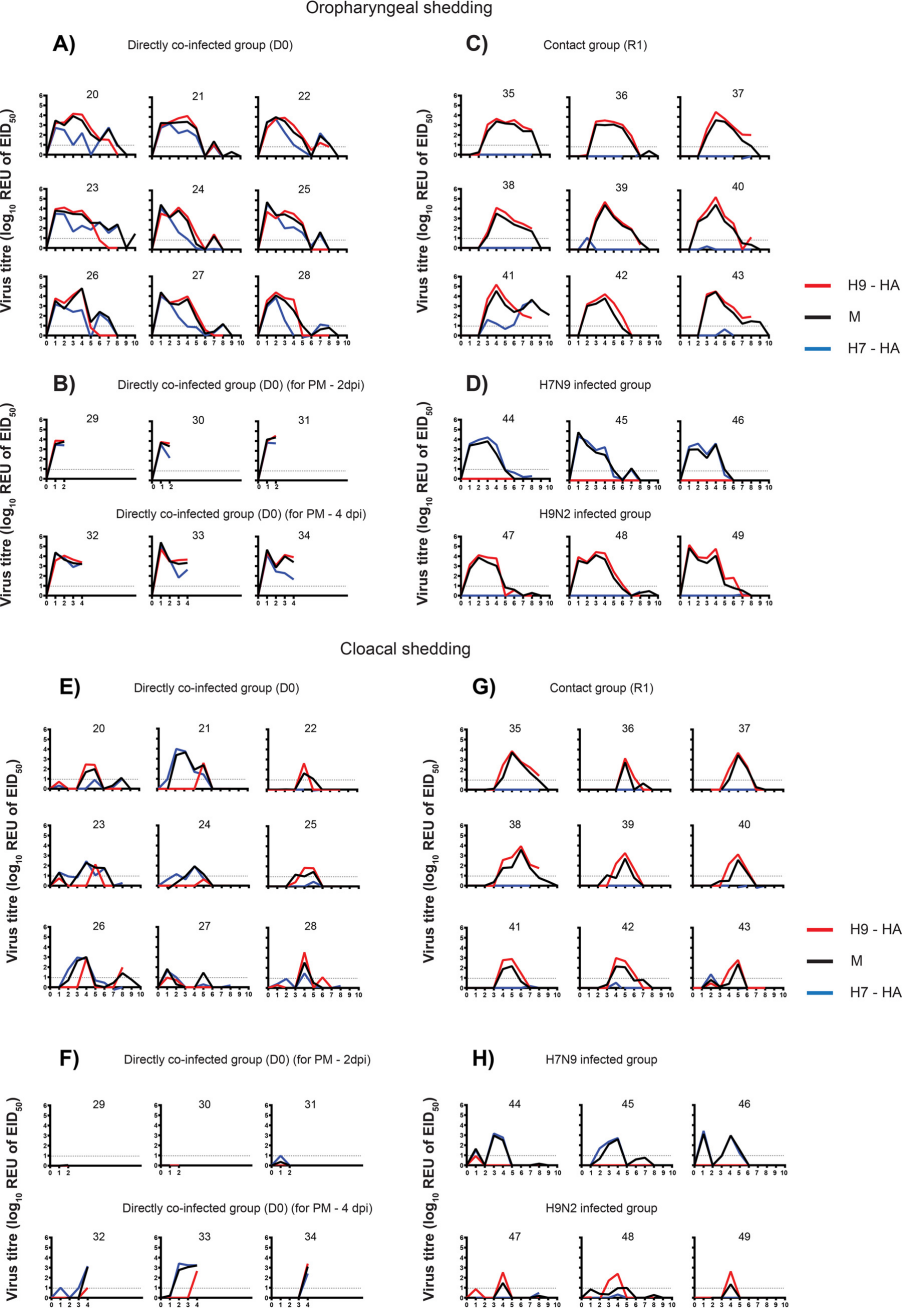
(A to H) Detection of oropharyngeal (A to D) and cloacal (E to H) shedding, together with HA and NA genotyping to identify the respective viral subtypes which had replicated in chickens. Influenza A viral RNA shedding profiles expressed as relative equivalence units (REUs of EID_50_) (oropharyngeal and cloacal, as indicated) are shown for the individual infected chickens (numbered in individual panels). A standard curve was constructed using a dilution series of Anhui/13 viral RNA extracted from a known infectious titer (EID50/mL) of the virus and tested by the M-gene H7- and H9-specific RT-qPCRs (see Materials and Methods), which were shown to be equivalent in assay performance (Fig. S1). The *C_T_* values were compared against an Anhui1/13 or UDL/08 RNA standard to determine relative equivalency units (REU of EID_50_). The dotted line represents the positive cutoff REU value. The chickens were directly coinfected with H9N2 UDL/08 virus and H7N9 Anhui/13 virus (A and E) to investigate transmission to contacts (C and G). Chickens were similarly coinfected and sacrificed at 2 and 4 dpi for virus dissemination in internal organs, with shedding similarly monitored (B and F). Shedding from chickens singly infected with either H7N9 Anhui/13 or H9N2 UDL/08 is also shown (D and H). The dotted horizontal line represents the REU of the EID_50_ value at the limit of positive viral detection. Influenza virus shedding in all chickens had ceased by 11 dpi, so viral titers at subsequent swabbing days are not shown.

Initially, we used three AIV RT-qPCRs (H7-RT-qPCR, H9-RT-qPCR, and the generic M-gene-RT-qPCR) to assess the oropharyngeal and cloacal shedding from chickens (i) directly coinfected with H7N9 and H9N2 (D0), (ii) placed in direct contact with the coinfected chickens (R1), (iii) directly infected with H7N9 only, and (iv) directly infected with H9N2 only (Fig. 1). In the transmission experiment, the M-gene RT-qPCR detected positive viral RNA shedding, from both oropharyngeal and cloacal swabs, in all nine D0 (directly infected) and all nine R1 (contact) chickens (Fig. 1A, C, E, and G), thereby demonstrating successful infection of the D0 chickens and transmission to all nine R1 contacts. Oropharyngeal shedding of viral RNA had declined and resolved by 8 to 9 days postinfection (dpi) for most of the chickens, except for no. 41, where shedding was still detectable at 10 dpi (Fig. 1A and C), and subsequently it was shown that all shedding had ceased by 11 dpi in all chickens (data not shown). However, cloacal shedding of viral RNA was less prominent, and at a generally lower titer, in the nine D0 chickens (Fig. 1E) compared to the oropharyngeal shedding but higher, and of more sustained duration, for the R1 contacts (Fig. 1G). All cloacal shedding in the D0 and R1 chickens had ceased by 9 dpi. Virus RNA shedding in the directly coinfected group (D0) kept for postmortem (PM) analysis at 2 dpi and 4 dpi showed viral RNA in the oropharyngeal samples, yet viral RNA could not be detected in the cloacal samples at 2 dpi (Fig. 1B and F).

Measurement of viral RNA shedding by the H7- and H9-specific RT-qPCRs in the singly infected chickens (Fig. 1D and H) showed the assays to yield very similar shedding results compared to the generic M-gene RT-qPCR, although some discrepancy was observed when shedding was at a low level. By applying the H7- and H9-specific RT-qPCR testing to the coinfected chickens, it was shown that in the D0 chickens, the oropharyngeal shedding of RNA from both subtypes was initially of a similar magnitude, but at later days of shedding the level of the H7 HA gene declined more rapidly, yet the shedding of the H9 HA gene remained at higher levels, i.e., comparable to that detected by the M-gene RT-qPCR (Fig. 1A). The overall lower level of cloacal shedding from the D0 chickens suggested either that H7 shedding declined prior to the H9 shedding or that H7 was weaker or below the positive threshold compared to the H9 shedding (Fig. 1E). Among the R1 contact chickens, detection of viral RNA in the oropharyngeal and cloacal cavities was, however, exclusively of the H9 subtype (Fig. 1C and G), the one exception being chicken no. 41, where H7 oropharyngeal shedding appeared to increase after H9 shedding had declined, although cloacal shedding in the same chicken was due to the H9 subtype alone. In summary, while early shedding in the D0 chickens showed both subtypes to be detectable among the total AIV progeny, shedding of the H9 subtype endured for longer, with the H9 subtype being preferentially transmitted to the R1 chickens.

### Emergence of H9N2 and H9N9 as dominant subtypes in coinfected chickens.

**FIG 2:**
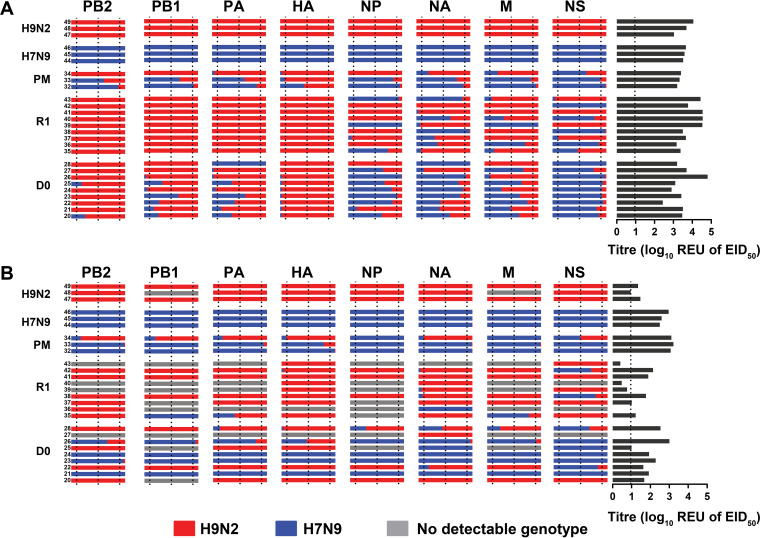
Genotyping of potential reassortant viruses shed from the oropharyngeal and cloacal cavities of D0 and R1 chickens at 4 dpi and 3 dpc, respectively. (A and B) RNA extracted from oropharyngeal (A) and cloacal (B) swab samples was used to quantify the proportions of each genetic segment using segment-specific RT-qPCRs for each parental virus strain (H9N2 UDL/08 shown in red; H7N9 Anhui/13 shown in blue). Swabs from individual chickens are represented in each horizontal row, with the proportions of each viral segment shown in separate columns. The *C_T_* values were compared against an Anhui1/13 or UDL/08 RNA standard to determine relative equivalency units (REU of EID_50_). The REU values obtained from the segment-specific RT-qPCRs were converted to illustrate the percentage frequency of the origins of each gene, shown by the relative lengths of the horizontal red and blue bars. The vertical dotted lines within gene segment columns represent 10%, 50%, and 90% frequencies of each gene. Annotation on the left denotes the *in vivo* infected chickens; H9N2 and H7N9 correspond to the single-infected control groups; PM corresponds to the chickens which were preplanned for cull and postmortem examination at 4 dpi (pathogenesis experiment); D0 and R1, respectively, indicate the direct- and contact-infected chickens following coinfection with both progenitor viruses; the final two digits of the individual chicken identifiers are discernible by the small font size at the left end of each row. On the right, the AIV REU of EID_50_ for each chicken’s swab is shown by dark gray horizontal lines, with the broken vertical line indicating the REU positive cutoff. The failure to detect the origins of a given viral genetic segment (shown by gray horizontal bars) among several cloacal swabs tended to occur in those with low viral shedding values.

**FIG 3:**
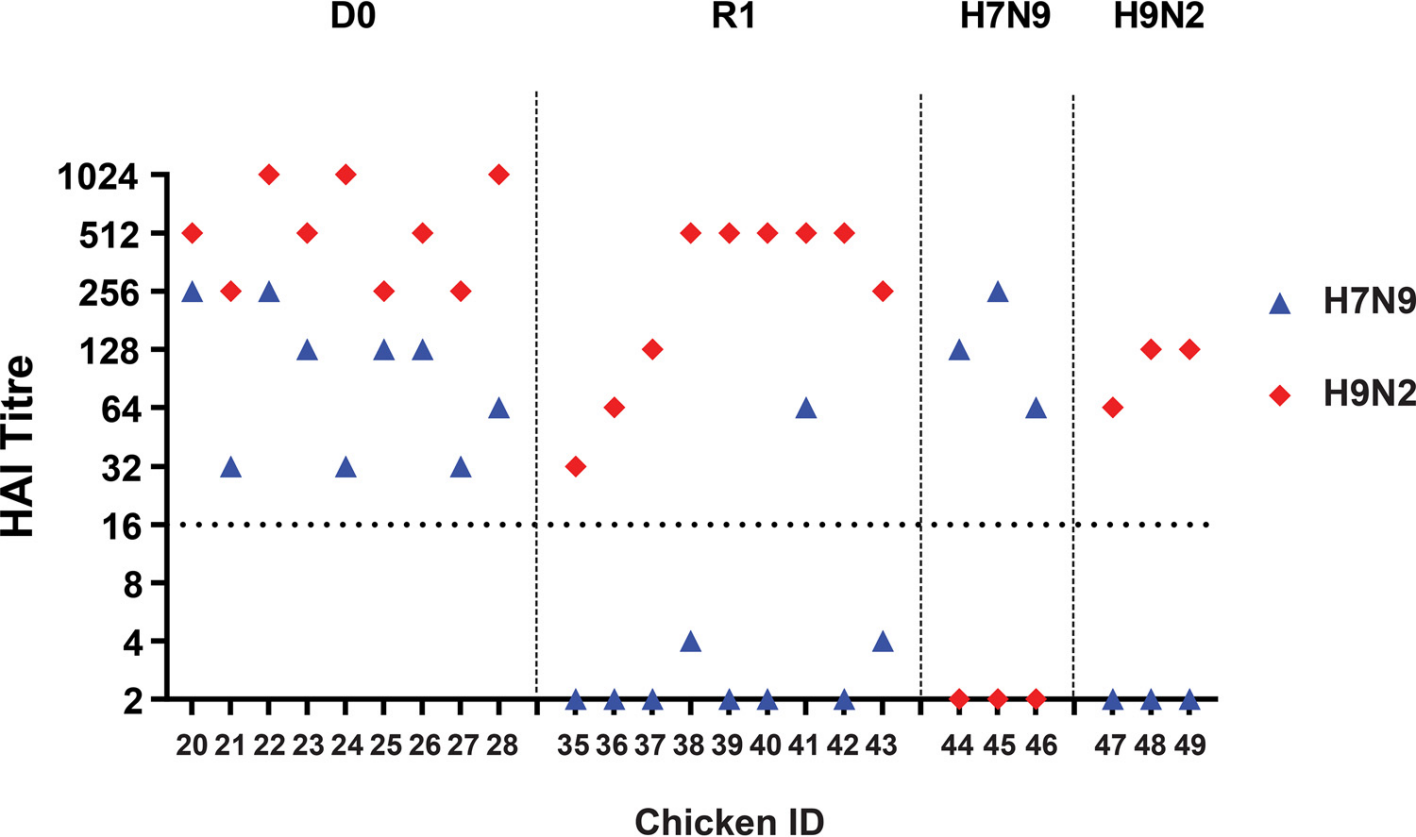
Seroconversion in chickens at 14 dpi/13 dpc demonstrated by the hemagglutination inhibition (HI) test. Seroconversion in chickens directly coinfected (D0), coinfected contacts (R1), or those singly infected with H7N9 (Anhui/13) or H9N2 (UDL/08) viruses, as indicated by the headers. Symbols represent chicken HI titers against H7N9 (blue) and H9N2 (red) homologous antigens. The broken horizontal line indicates the HI positive cutoff value of 16 hemagglutination units. The 14 dpi time point for D0 chickens corresponds to the 13 dpc time point for the R1 chickens because the latter were introduced at 1 dpi.

From the preliminary analysis with the H7, H9, and generic M-gene-based RT-qPCRs, 4 dpi was selected as the time point where oropharyngeal viral RNA appeared to be maximal, or near maximal, for the majority of the coinfected D0 and R1 chickens (Fig. 1). Therefore, swabs from 4 dpi were selected to distinguish the origins (Anhui/13 or UDL/08) of all eight AIV genetic segments by using the segment-specific RT-qPCRs (Table S1). Many of the 4-dpi swabs (oropharyngeal and cloacal) among the D0 chickens possessed a mixture of segments of both Anhui/13 and UDL/08 origins, although the UDL/08 origin H9 gene was strongly dominant among the HA segments analyzed from the oropharyngeal swabs (Fig. 2A). Cloacal shedding of unreassorted Anhui/13 in D0 chickens was restricted to only three D0 birds (chickens no. 21, 23, 24) (Fig. 2B). Similar predominant cloacal shedding of Anhui/13 was observed in two chickens (no. 32 and 33) which were sacrificed for postmortem examination at 4 dpi. There was only one instance of H7 segment transmission to one R1 chicken (no. 41) (Fig. 1C), affirmed as the only example of H7 seroconversion among the R1 chickens (Fig. 3).

However, among the R1 chickens, contact transmission had resulted in an altered preponderance of UDL/08-origin segments, particularly for the HA (H9) and the three polymerase genes (PB2, PB1, and PA) among the R1 oropharyngeal swabs where no corresponding Anhui/13-origin segments were detected at all (Fig. 2A). These R1 oropharyngeal swabs included a variety of potential reassortants (genotypes), as evidenced by various proportions of the mixed origins of the nucleoprotein (NP), NA, M, and NS genes. Therefore, a mix of novel genotypes (which included the H9N2 and H9N9 subtypes) appear to have emerged (or were in the process of emerging) from the oropharynx of the R1 chickens. The reassortant H7N2 subtype may have consisted of a small minority viral population at the D0 stage which failed to transmit to the R1 chickens (Fig. 2). Overall, these results indicated a stronger replicative and transmissible fitness for the HA and polymerase gene segments of UDL/08 H9N2 virus origin.

### Virus dissemination in the D0 coinfected chickens.

To address virus dissemination in chickens coinfected with H7N9 and H9N2 viruses, three chickens were sacrificed at 2 dpi and at 4 dpi. M-gene RT-qPCR revealed the highest viral RNA load in nasal turbinates (estimated as log_10_ relative equivalence units [REU] of 50% embryo infectious dose [EID_50_]) compared to all the tissues (Fig. S2). The viral RNA levels in the nasal turbinates were significantly higher at 2 dpi (1.85 × 10^4^ REU of EID_50_) compared to 4 dpi (1.91 × 10^3^ REU of EID_50_) (*P* < 0.05), but there was no evidence of detectable infection in other organs within the respiratory tract (data not shown). The next highest viral loads were detected in the brain (1.08 × 10^3^ REU of EID_50_), followed by the cecal tonsils (3.43 × 10^2^ REU of EID_50_), but the difference was not significant between 2 dpi and 4 dpi (*P* > 0.05), although RNA levels were higher in brain on 4 dpi. Several other organs revealed very low or subthreshold levels (<1 × 10^1^ REU of EID_50_) of viral infection.

### Seroconversion in D0 coinfected chickens and the R1 contacts.

Serum samples were collected at 14 dpi from coinfected (D0) and contact (R1) chickens and tested for antibodies by hemagglutination inhibition (HI) assay. The three single-infected control chickens seroconverted by 14 dpi to their respective homologous H7 or H9 subtype antigen (Fig. 3). The nine coinfected D0 chickens showed a stronger seroconversion in the form of anti-H9 than anti-H7 antibodies at the same 14-dpi time point. Eight of nine (89%) R1 contacts bled at the same time (13 days postchallenge [dpc]) reacted to only the H9 subtype antigen, with R1 chicken no. 41 showing HI titer against H9 antigen but registering a weaker seroconversion against the H7 antigen (Fig. 3). The viruses detected from this chicken (no. 41) included both the H7 and H9 HA segments during oropharyngeal shedding (Fig. 1C).

### The ribonucleoprotein complex of H9N2 displays higher polymerase activity compared to that of H7N9 in chicken cells.

**FIG 4:**
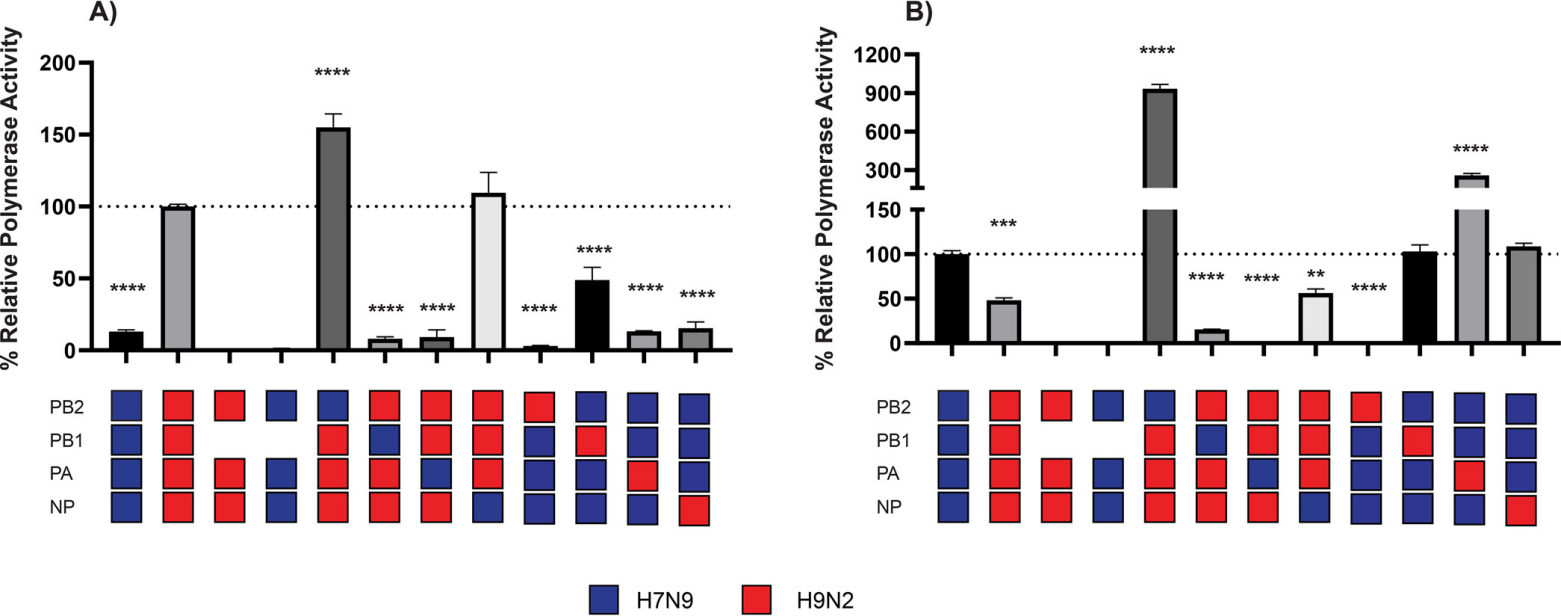
Minireplicon assay of the ribonucleoprotein (RNP) complexes of H7N9 Anhui/13 and H9N2 UDL/08 and four mixed RNP combinations. (A and B) The RNP gene complexes derived from Anhui/13 and UDL/08 viruses were reconstituted by transfection of (A) chicken DF-1 and (B) human HEK-293T cells, along with four mixed RNP combinations from the H7N9 and/or H9N2 viruses. The cells were incubated at 37°C (HEK-293T) or 39°C (DF-1). At 24 h posttransfection, cells were lysed, and luciferase activities were measured. Cotransfection of plasmids without PB1 served as a negative control for RNP activity. Polymerase activity of H9N2 in DF-1 cells and H7N9 in HEK-293T was set at 100%, and the percent (%) relative polymerase activity was calculated. The data shown are representative of three independent experiments and are shown as the mean percent relative polymerase activity with error bars showing the standard error of mean (SEM) of three different replicates. ****, *P* < 0.0001; ***, *P* < 0.0005; **, *P* < 0.005.

An increase or decrease in polymerase activity can also be linked with the replication fitness or adaptability of a reassortant virus in a target host. As we previously observed enrichment of UDL/08 polymerase genes in the reassortant viruses, we therefore investigated ribonucleoprotein (RNP) activity of polymerase gene segments of reassortant viruses using a minireplicon assay. We quantified the polymerase activity of different RNP combinations obtained from the parental UDL/08 (H9N2) or Anhui/13 (H7N9) viruses. The RNP complex consisting of all four genes (PB2, PB1, PA, and NP) from UDL/08 H9N2 showed more polymerase activity (*P* < 0.0001) compared to Anhui/13 H7N9 RNP in chicken DF-1 cells (Fig. 4A). Inclusion of the Anhui/13-origin PB2 produced greater polymerase activities compared to the unaltered UDL/08 RNP in chicken cells (*P* < 0.0001). Including H7N9-origin PB1 or PA on the UDL/08 background reduced the activity compared to that of the unaltered UDL/08 RNP (*P* < 0.0001), while UDL/08 PB1 increased the polymerase activity on the Anhui/13 background (*P* = 0.0091) compared to Anhui/13 RNP in DF-1. These results showed that the RNP complex of UDL/08 has a greater polymerase activity compared to the RNP complex of Anhui/13 virus in DF-1 cells (*P* < 0.0001), with this greater activity attributed, at least, to the UDL/08 PB1 and PA gene segment.

The RNP complex of UDL/08 H9N2, on the other hand, showed a significantly lower polymerase activity compared to Anhui/13 H7N9 RNP in human HEK-293T cells (*P* < 0.0001) (Fig. 4B). Inclusion of PB2 from Anhui/13 H7N9 on the UDL/08 H9N2 background significantly increased the polymerase activity (*P* < 0.0001) compared to Anhui/13 (Fig. 4B). PB1 and PA from Anhui/13 H7N9 further reduced the polymerase activity, and NP from Anhui/13 H7N9 resulted in a similar polymerase activity on the UDL/08 H9N2 background. PA from UDL/08 H9N2 increased the polymerase activity (*P* < 0.0001) on the Anhui/13 background compared to unaltered Anhui/13 RNP. These results suggest that the higher polymerase activity of Anhui/13 H7N9 in human cells is attributed mainly to the PB2 gene segment.

### Multistep replication kinetics and 50% egg lethal dose.

**FIG 5:**
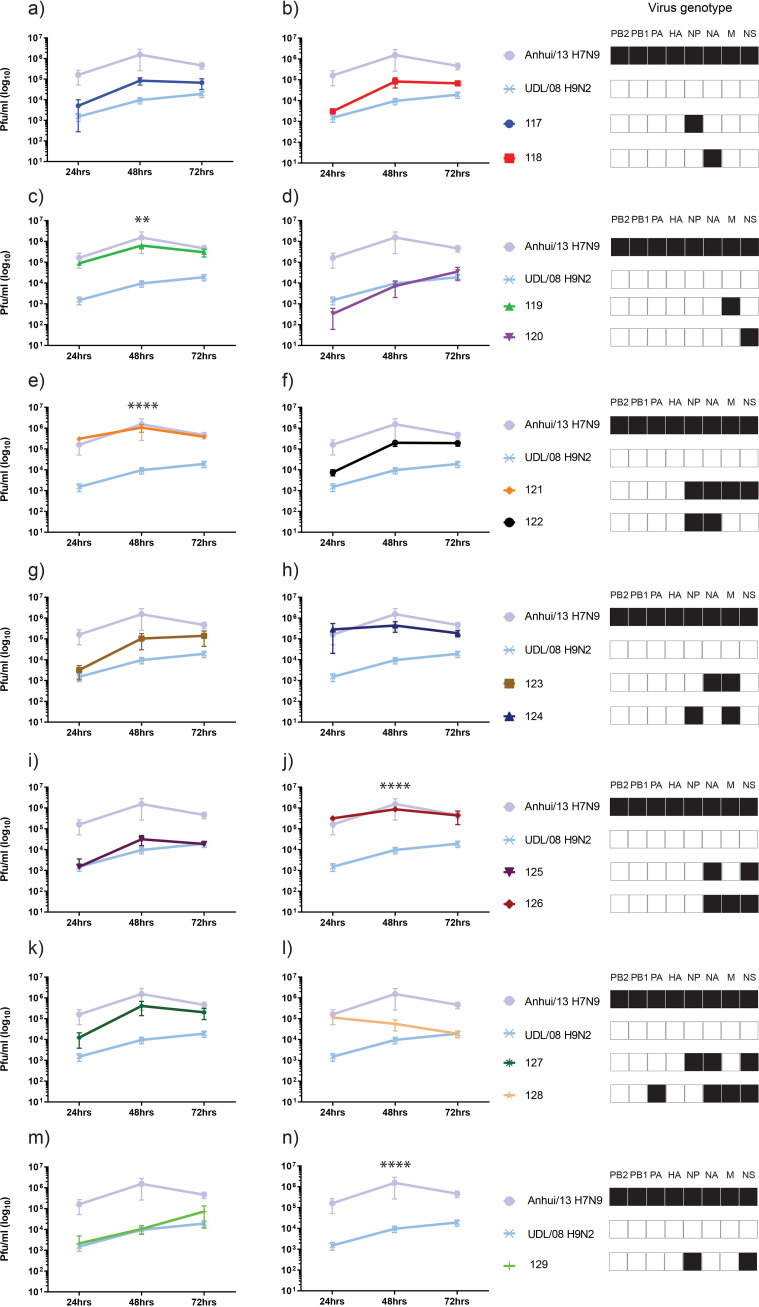
Multistep replication kinetics of reassortant H9Nx viruses in primary chicken kidney (CK) cells. Primary CK cells were infected with 0.0002 multiplicity of infection (MOI) of the 13 H9Nx reassortants or either of the parental virus strains. Cell supernatants were harvested at 24 h, 48 h, and 72 h postinfection and titrated by plaque assay. Each time point corresponds to the mean of four biological replicates with standard deviations indicated. Replication kinetics of each reassortant virus compared to parental Anhui/13 H7N9 and UDL/08 H9N2 viruses is shown in panels a to n. The genotype of each reassortant virus is shown as a combination of black and white boxes, with black indicating Anhui/13 H7N9 origin and white indicating UDL/08 H9N2 origin gene segments. **, *P* < 0.005; ****, *P* < 0.0001 compared to UDL08 H9N2.

**FIG 6:**
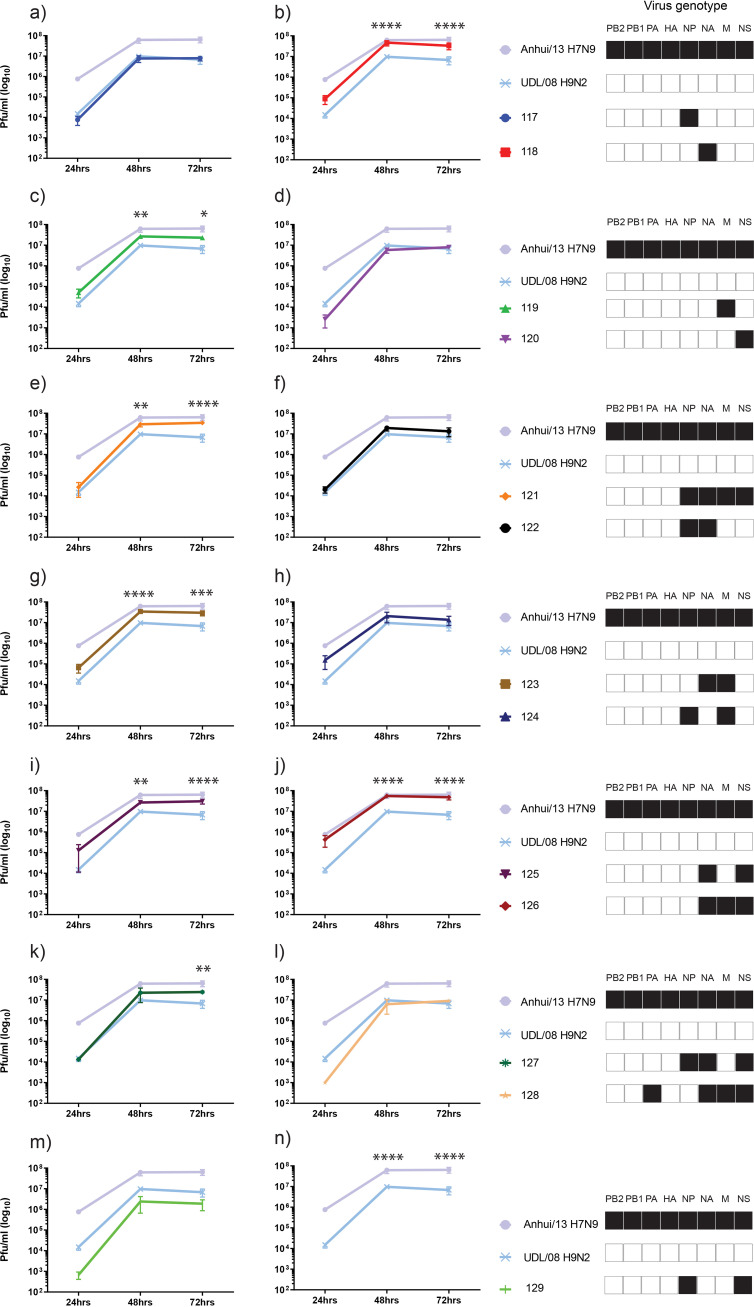
Multi-step replication kinetics of reassortant H9Nx viruses in Madin Darby canine kidney (MDCK) cells. MDCK cells were infected with 0.0002 multiplicity of infection (moi) of the 13 H9Nx reassortants and both parental virus strains. Cell supernatants were harvested at 24hr, 48hr and 72 hr post-infection and titrated by plaque assay. Each time point corresponds to the mean of four biological replicates with standard deviations indicated. Replication kinetics of each reassortant virus compared to parental Anhui/13 H7N9 and UDL/08 H9N2 viruses is shown in panels (a) to (n). The genotype of each reassortant virus is shown as a combination of black and white coloured boxes with black indicating Anhui/13 H7N9 origin and white indicating UDL/08 H9N2 origin gene segments. * denotes *P* value <0.05, ** denotes *P* value <0.005 and **** denotes *P* value <0.0001 compared to UDL/08 H9N2.

**FIG 7:**
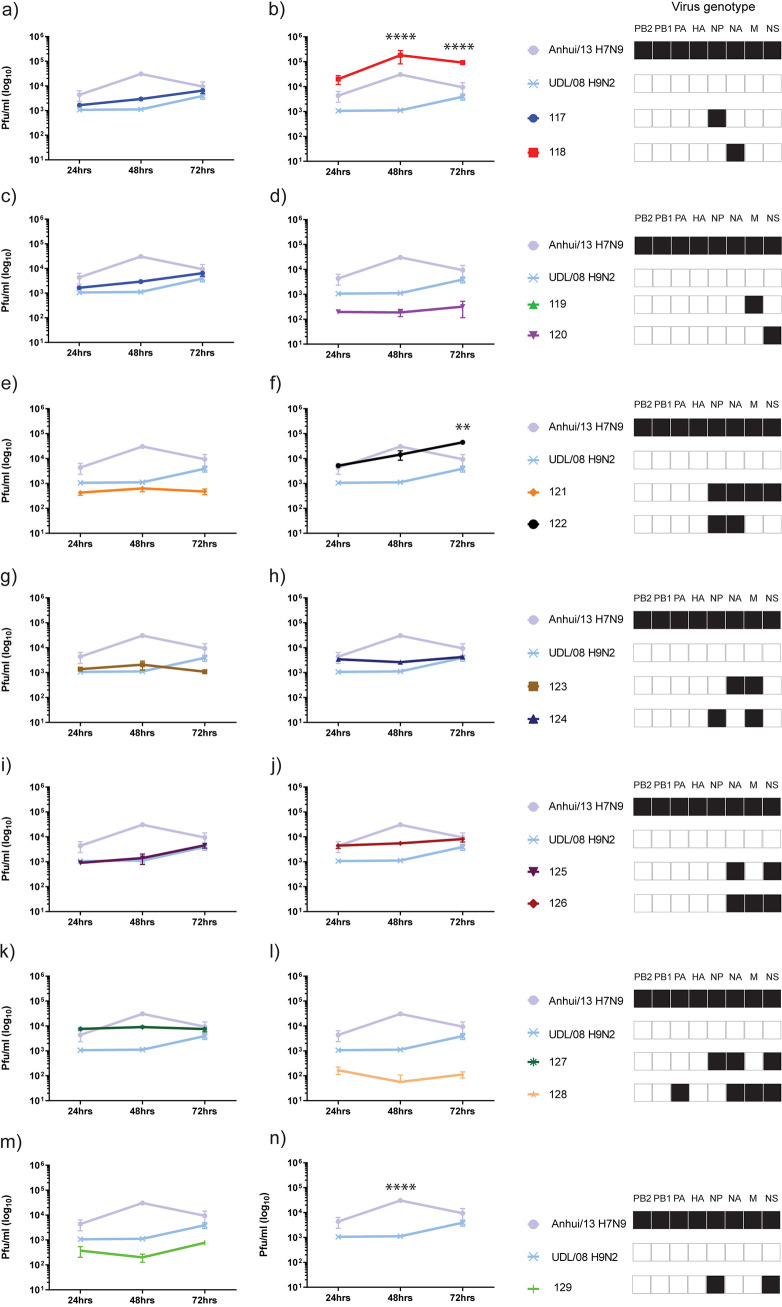
Multi-step replication kinetics of reassortant H9Nx viruses in human lung epithelial (A549) cells. A549 cells were infected with 0.05 multiplicity of infection (moi) of each reassortant and both parental virus strains. Cell supernatants were harvested at 24hr, 48hr and 72 hr post-infection and titrated by plaque assay. Each time point corresponds to the mean of four biological replicates with standard deviations indicated. Replication kinetics of each reassortant virus compared to parental Anhui/13 H7N9 and UDL/08 H9N2 viruses is shown in panels (a) to (n). The genotype of each reassortant virus is shown as a combination of black and white coloured boxes with black indicating Anhui/13 H7N9 origin and white indicating UDL/08 H9N2 origin gene segments. ** denotes *P* value <0.005 and **** denotes *P* value <0.0001 compared to UDL/08 H9N2.

Based on the RT-qPCR data (Fig. 2), the gene segments contributed by parental UDL/08 H9N2 and Anhui/13 H7N9 toward the reassortant viruses which could have formed and shed from the oropharynx and cloaca of R1 chickens were identified and attempted for virus rescue by reverse genetics (RG). These included 11 genotypes (117 to 127) from the oropharyngeal swab samples and three (128, 129, and 130) from cloacal swabs (Table 1). The reassortant viruses and two parental strains were compared for their plaque-forming ability (Fig. S3) and chicken embryo lethality (Table 1) along with replication in primary chicken kidney cells (CK) (Fig. 5), MDCK cells (Fig. 6), and human A549 cells (Fig. 7). For the replication of progenitor strains in CK cells, Anhui/13 H7N9 clearly displayed significantly greater kinetics than UDL/08 at 48 h postinfection (*P* < 0.0001) (Fig. 5n, but included in all panels). Acquisition of the M gene from Anhui/13 either alone (genotype 119) (Fig. 5c), in combination with NP, NA, and NS (genotype 121) (Fig. 5e), or with NA and NS (genotype 126) (Fig. 5j) gene segments from H7N9 substantially increased the replication of reassortant viruses at 48 h postinfection compared to UDL/08.

**TABLE 1:** Genotypes of the potential reassortant viruses in swabs samples which emerged after coinfection and transmission to R1 chickens^*a*^

Genotype ID	Virus genotype								Egg lethal dose (ELD_50_) (pfu/mL)
	PB2	PB1	PA	HA	NP	NA	M	NS	
117									>1.00E+06
118									2.29E+02
119									1.55E+03
120									>1.00E+05
121									6.46E+02
122									2.40E+02
123									>1.00E+05
124									1.00E+02
125									>1.00E+05
126									>1.00E+05
127									>1.00E+05
128									>1.00E+05
129									>1.00E+05
130									Did not rescue
Anhui/13									2.51E+03
UDL/08									2.14E+03
		H9N2 origin gene segment					H7N9 origin gene segment		

a Oropharyngeal and cloacal swab samples from the contact chickens (R1) were processed, and potential reassortant viruses were identified by RT-qPCR as shown by virus genotype across each genotype ID. The viruses were rescued by reverse genetics and compared for their 50% egg lethal dose (ELD_50_) in 10-day-old SPF embryonated eggs. The genotypes 117 to 127 were identified from oropharyngeal samples, while 128 to 130 were identified in the cloacal swab samples. The dark grey shading denotes H7N9 origin gene segments and light grey shading denotes H9N2 origin gene segments.

The viral replication kinetics in MDCK cells showed significantly higher replication for the Anhui/13 progenitor at 48 h and 72 h postinfection compared to the UDL/08 progenitor (*P* < 0.0001) (Fig. 6n, but included in all panels). Further, acquisition of the NA or M gene segment from H7N9 substantially increased the replication of reassortant genotypes 118 and 119, respectively, compared to UDL/08 H9N2 virus (Fig. 6b and c). In human lung A549 cells, the replication kinetics of progenitor Anhui/13 was again significantly greater at 48 h postinfection compared to the UDL/08 progenitor (*P* < 0.0001) (Fig. 7n, but included in all panels). However, the reassortant virus with NA of Anhui/13 origin, namely, genotype 118 (H9N9), showed significantly higher replication compared to both progenitor H7N9 and H9N2 viruses at 48 and 72 h postinfection (*P* < 0.0001) (Fig. 7b). The reassortant H9N9 genotype 122 (having NP and NA of H7N9) also showed significantly higher replication compared to H7N9 and H9N2 viruses at 72 h postinfection (*P* < 0.005) (Fig. 7f). Genotype 122 also displayed increased replication kinetics relative to UDL/08 in both CK and MDCK cells, albeit without statistical significance (*P* > 0.05) (Fig. 5f and 6f).

Five reassortant viruses also showed increased embryo lethality (lower embryo lethal dose (ELD_50_) compared to the progenitor viruses (Table 1). These five genotypes had acquired various segments from the Anhui/13 progenitor, namely, (i) the genotype 118 H9N9 virus which acquired the NA gene; (ii) the genotype 119 H9N2 virus which acquired the M gene; (iii) the genotype 121 H9N9 virus which acquired NP, NA, M, and NS; (iv) the genotype 122 H9N9 virus which acquired NP and NA; and (v) the genotype 124 H9N2 virus which acquired NP and M. These observations suggest that the M and NA genes of the H7N9 virus, when present as 1+7 gene combinations with H9N2, enable greater adaptability for avian and mammalian hosts, respectively, as reflected in generally increased *in vitro* replication fitness. However, the contribution of M or NA on increased replication of 1+7 reassortant viruses reduces when present in combinations with other Anhui/13 genes.

### Plaque purification of viruses from R1 chicken swabs to identify viable reassortant viruses.

Following initial coinfection of the D0 chickens, AIV RNA shedding at relatively high titers at 4 dpi (3 dpc) from the oropharyngeal cavity of the R1 chickens represented virus(es) that were sufficiently fit and had successfully transmitted within this host. To elucidate the exact constellation of genes within any viable reassortant virus(es), plaque purification of the oropharyngeal swab samples from all the nine R1 contact chickens was carried out in MDCK cells. Discrete plaques between a range of 16 and 28 were isolated, and RNA was extracted to fully characterize their genotype by segment-specific RT-qPCRs. The genotype frequencies in each sample (expressed as a percentage [%]) were calculated by taking the ratio of the number of times a particular genotype appeared by total plaques isolated for a particular sample (Table 2). The genotypes identified by plaque purification reflected the overall genotyping as identified by RT-qPCR of swab samples (Fig. 2). However, not all genotypes identified by RT-qPCR of swab samples could be isolated by plaque purification. The unreassorted H9N2 UDL/08 was detected in four out of nine chickens between 100% and 13.6% genotype frequencies. In addition, a total of eight novel genotypes, including single, double, and triple segment reassortants, were detected. All the viruses (Table 2) which had the highest genotype frequency (marked as bold) included genotype 120 (7+1 reassortant H9N2 with NS from Anhui H7N9; isolated from four chickens at 100%, 12.5%, 4.2%, and 3.7% genotype frequencies), followed by genotype 125 (6+2 reassortant H9N9 with NA plus NS from Anhui H7N9; isolated from two chickens at 95.8% and 9.5% genotype frequency), genotype 122 (6+2 reassortant H9N9 with NP plus NA from H7N9; isolated from two chickens at 88.9% and 12.5% genotype frequency), and genotype 124 (6+2 reassortant H9N2 with NP plus M from H7N9; isolated from one chicken at 100% genotype frequency). UDL/08 H9N2 virus contributed substantially in terms of gene segments for different reassortant viruses and, interestingly, the H9N2 HA and polymerase gene segments (PB2, PB1, and PA) were conserved in 100% of the plaque isolates analyzed in the R1 contact chickens (Table 2).

**TABLE 2:** Genotypes of the reassortant viruses isolated after plaque purification from the oropharyngeal swab samples from the coinfected contact chickens at 3 dpc

Genotype ID	Chicken ID	Genotype frequency (%) (no. of plaques)^*a*^	Virus genotype							
			PB2	PB1	PA	HA	NP	NA	M	NS
UDL/08	41	**100 (24)**								
	40	66.7 (14)								
	37	56.3 (9)								
	36	13.6 (3)								
120	42	**100 (23)**								
	37	12.5 (2)								
	38	4.2 (1)								
	43	3.7 (1)								
119	36	68.2 (15)								
	43	7.4 (2)								
	40	4.8 (1)								
125	38	**95.8 (23)**								
	40	9.5 (2)								
122	43	**88.9 (24)**								
	37	12.5 (2)								
118	40	19 (4)								
	36	18.2 (4)								
117	37	12.5 (2)								
124	39	**100 (28)**								
127	37	6.3 (1)								
				H9N2 origin gene segment				H7N9 origin gene segment		

a The genotype frequencies in each sample were calculated by taking the ratio the of number of times a particular genotype appeared in each sample by the total plaques isolated for a particular sample expressed as a percentage. The bold values indicate the predominant genotypes identified. The dark grey shading denotes H7N9 origin gene segments and light grey shading denotes H9N2 origin gene segments.

Comparison of the percentage genotype frequency of reassortant viruses (Table 2) and their replication in human A549 cells (Fig. 7) showed that genotype 122 notably had higher a genotype frequency and showed increased replication. All the other reassortant genotypes which had a higher genotype frequency (genotypes 120, 124, and 125) were attenuated for their replication in A549 cells. These results indicated that out of all the predominant genotypes, genotype 122 showed increased replication in human A549 cells. This observation warranted further investigation with respect to the zoonotic potential of genotype 122 H9N9 virus.

### Reassortant H9N9 virus has preferential receptor binding to α2,6 sialic acid receptor analogues compared to the parental UDL/08 (H9N2 virus).

**FIG 8:**
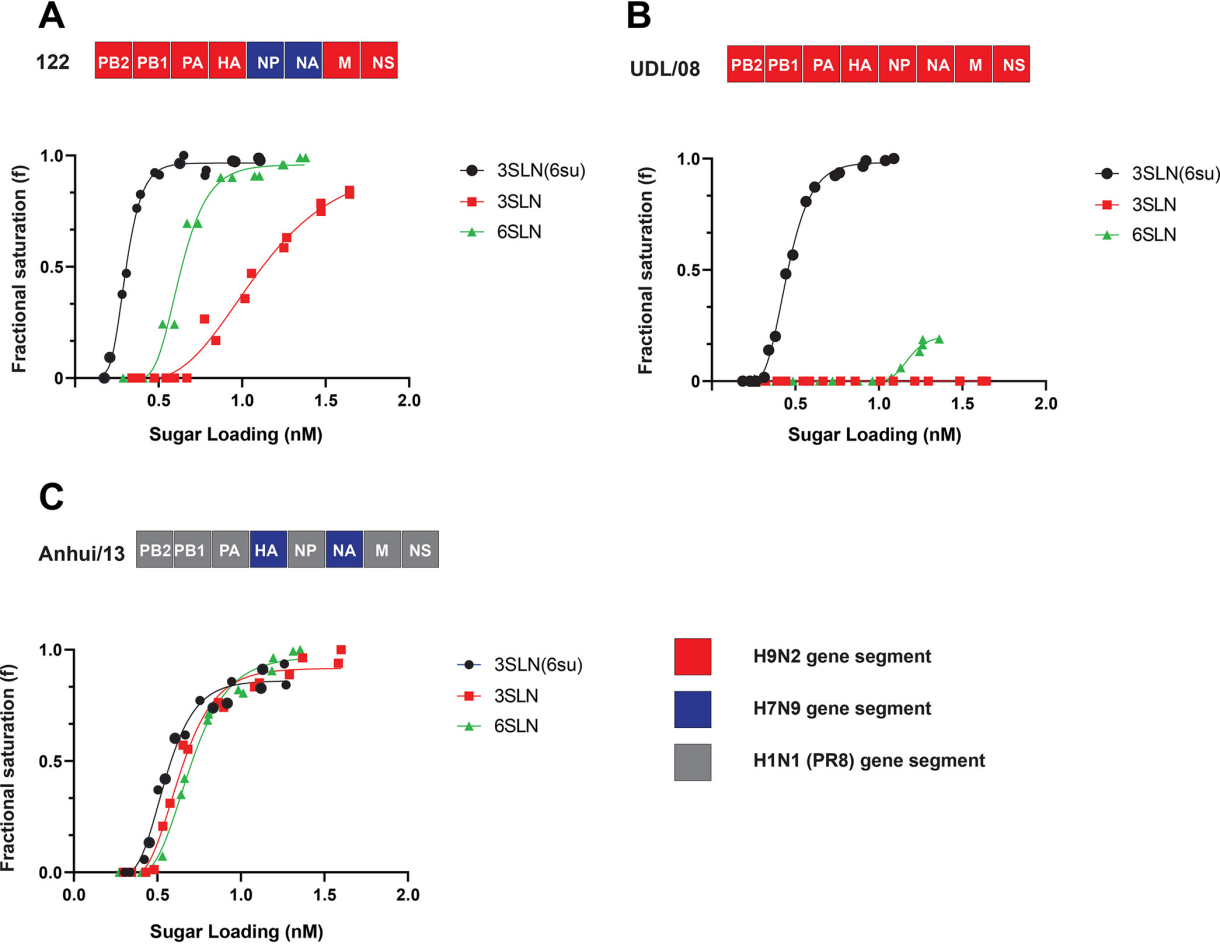
Receptor binding profiles of reassortant H9N9 virus compared to H9N2 and H7N9 viruses. (A) Binding of 6+2 reassortant H9N9; genotype 122 (two genes [NP and NA] from Anhui/13 H7N9 and 6 genes from UDL/08 H9N2) to α-2,3-linked (3′SLN 6-sulfated) (black), α-2,3-linked (3′SLN) (red), or α-2,6-linked (6′SLN) (green) sialylglycan receptors was determined by biolayer interferometry. (B and C) Similar receptor binding profiles were determined for (B) UDL/08 H9N2 and (C) 2+6 reassortant H7N9 (2 genes [HA and NA] from Anhui/13 H7N9 plus 6 genes from PR8). Since biolayer interferometry involved testing of infectious virus, due to biosafety reasons, the receptor binding of H7N9 was carried out using the 2+6 reassortant of H7N9, which included internal genes from PR8.

Host receptor binding preference of influenza viruses is a critical determinant of host adaptation and airborne transmission in ferrets (43). The receptor binding specificity of parental and reassortant H9N9 viruses were quantified with synthetic sialoglycopolymers—α2,6-sialyllactosamine (6SLN), α2,3-sialyllactosamine (3SLN), or Neu5Ac α2,3Gal β1-4(6-HSO_3_) GlcNAc (3SLN [6-su]), receptor analogues using bio-layer interferometry (44). The selected reassortant H9N9 virus (genotype 122, Fig. 8A) showed strong binding for 6SLN receptors which was comparable to that of Anhui/13 (Fig. 8C). In contrast, the parental UDL/08 displayed only marginal and undetectable binding to6SLN receptor analogues (Fig. 8B). The reassortant H9N9 viruses also bound strongly to 3SLN compared to the parental UDL/08 H9N2 virus, which showed no binding (Fig. 8A and B). The binding avidity for 3SLN (6-Su) was also stronger for the reassortant H9N9 viruses compared to the UDL/08 H9N2 virus. These observations showed that genotype 122 H9N9 virus has increased receptor binding preferences for both avian and mammalian hosts compared to the genetic donor viruses.

### Fusion pH of reassortant H9N9 virus compared to parental Anhui/13 and UDL/08.

The pH of fusion critically influences stability and infectivity of virus in the target host species. The viruses that are stable at lower pH carry greater propensity to retain infectivity in the human airway epithelium. We determined the fusion pH of the reassortant H9N9 virus (genotype 122) and the parental H7N9 and H9N2 viruses in Vero cells using a syncytium-formation assay. For H7N9 virus infections, cells showed optimal pH fusion at 5.6, while for reassortant H9N9 virus (genotype 122) and the parental H9N2 virus, infected cells demonstrated optimal fusion at pH 5.4. This observation showed that the reassortant H9N9 virus has a greater pH stability of the HA compared to that of the H7N9 viruses.

### Assessment of the zoonotic risk of the selected reassortant H9N9 virus.

**FIG 9:**
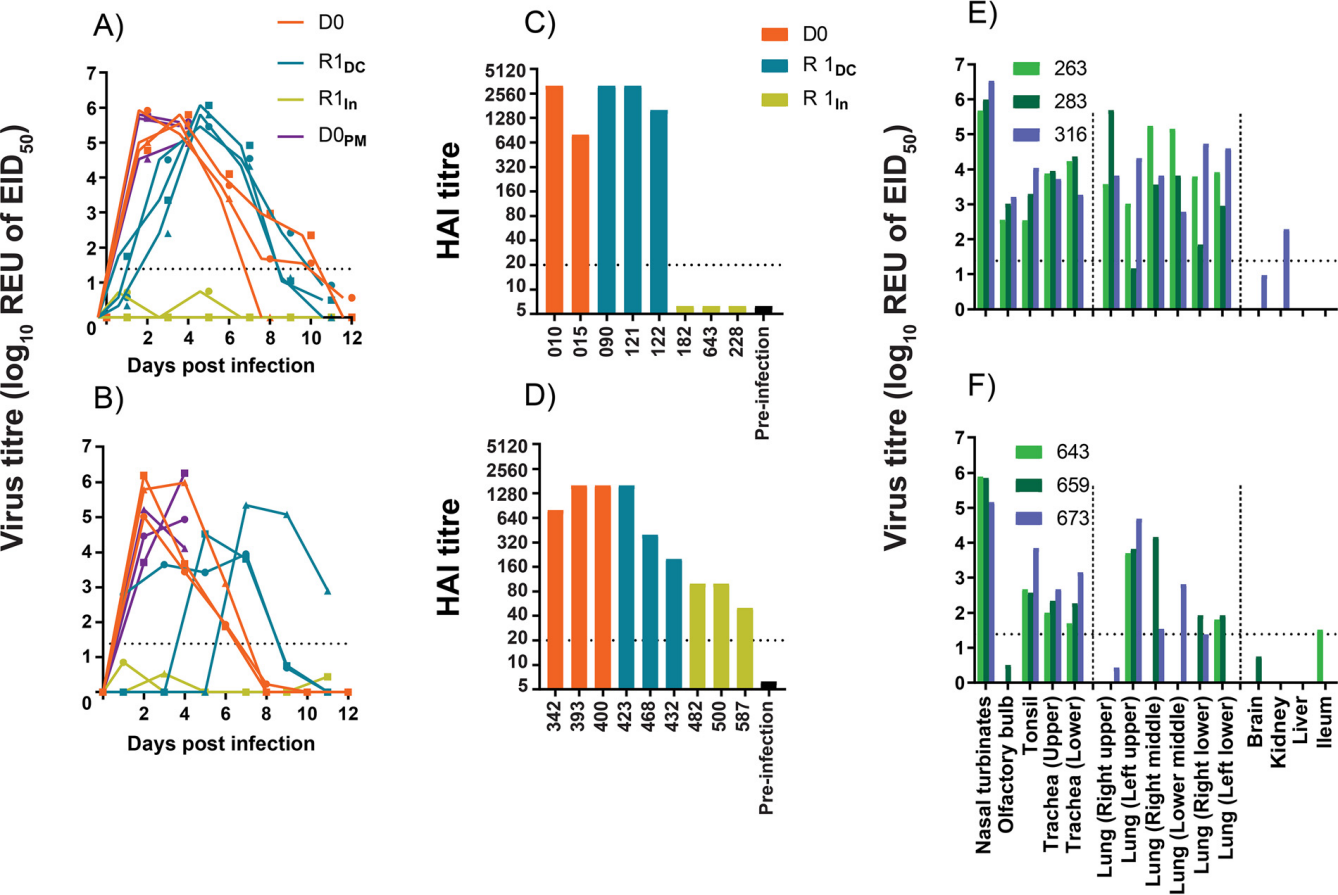
Detection of influenza A virus RNA and seroconversion in ferrets after intranasal inoculation with H7N9 and reassortant H9N9 viruses. (A and B) Two separately housed groups of ferrets (D0, *n* = 6 per group) were infected directly via the intranasal route with (A) Anhui/13 and (B) the reassortant H9N9 (genotype no. 122). At 1 dpi, a group of ferrets (*n* = 3) was placed in direct contact (R1_DC_) with each group of D0 ferrets, while another indirect contact group (R1_In_) of ferrets (*n* = 3) was placed in an adjacent cage. Nasal washes were collected on alternate days from all ferrets which remained in the study until 12 dpi to determine viral shedding (REUs of EID_50_) by the M-gene RT-qPCR. Along the horizontal axis, dpi corresponds to time points following the initial direct-infection of the D0 ferrets. (C and D) Seroconversion in D0, R1_DC_, and R1_In_ ferrets at 14 dpi/13 dpc. One ferret from the D0 group (H7N9) had to be euthanized at 6 dpi on welfare grounds. Thus, no HAI could be performed on this ferret. (E and F) Three ferrets from each D0_PM_ group were euthanized for postmortem at 4 dpi to assess virus dissemination in internal organs by M-gene RT-qPCR. The broken horizontal line corresponds to the positive cutoff value for the M-gene RT-qPCR (A, B, E, and F) and the HI (C and D) tests.

We further assessed the zoonotic potential of the novel H9N9 genotype using ferrets as an animal model of infection in humans. The findings from the quantitative investigations of the viruses shed from the oropharynx of chickens along with the replication kinetics, receptor binding, and fusion pH guided the assessment of genotype 122 (H9N9 reassortant) and its comparison to Anhui/13 H7N9 virus in a ferret transmission study. The ferrets directly infected (D0) with Anhui/13 H7N9 were positive for virus RNA detected in the nasal wash samples from 2 to 10 dpi, while the D0 ferrets infected with H9N9 showed positive shedding from 2 to 6 dpi (Fig. 9A and B). However, the peak level virus shedding was comparable in both D0 groups. Both the Anhui/13 H7N9 virus and reassortant H9N9 (genotype 122) virus exhibited 100% transmission efficiency from ferret to ferret when in direct contact (R1_DC_); all R1_DC_ contact ferrets became infected and shed virus from the nasal cavity (Fig. 9A and B). However, ferrets sharing the same airspace but separated physically (indirect) via a dividing mesh in adjacent cages did not show detectable viral shedding in either group (R1_In_).

All directly infected (D0) and direct-contact (R1_DC_) ferrets seroconverted when tested by HI assay against homologous viruses (Fig. 9C and D). None of the ferrets indirectly exposed to the Anhui/13 H7N9 virus-infected group (R1_In_) seroconverted to H7N9 virus, but all ferrets indirectly exposed to the reassortant H9N9 virus-infected ferrets (R1_In_) seroconverted to H9N9 virus (Fig. 9C and D).

Three ferrets in each of the D0 groups were culled at 4 dpi in order to provide a range of organs, mainly from the respiratory tract, for postmortem examination. Both the Anhui/13 and reassortant H9N9 viral RNA were detected at high levels (>5log_10_ REU of EID_50_) in the nasal turbinates of the D0 ferrets, with the former also detected in the olfactory lobe (Fig. 9E and F). Viral nucleoprotein was detected in the respiratory and olfactory epithelium of the nasal turbinates in all three infected ferrets (Fig. S4). In addition, histological lesions were identified in the respiratory epithelium of all ferrets for both viruses and, to a lesser extent, in the olfactory epithelium for both viruses (Fig. S4). Viral RNA for both viruses was detected in the upper and lower trachea; however, viral nucleoprotein antigen was not detected by immunohistochemistry (IHC) in the trachea in the H9N9-infected group (Table S2). Anhui/13 virus RNA was detected in the upper, middle, and lower lung tissues, whereas the reassortant H9N9 virus RNA was higher in the upper-left lobe of the lungs, with sporadic detection in the other lobes (Fig. 9E and F and Table S2). Overall, both H7N9 and H9N9 replicated in the nasal turbinates of directly infected ferrets. H7N9-infected ferrets showed more pulmonary lesions compared to H9N9-infected ferrets (Fig. S5). Neither the H7N9 or H9N9 viruses was detected in the brain or liver, although Anhui/13 was detected at a low level (>2log10 REU of EID_50_) in the kidney of one infected ferret (Fig. 9E and F), while H9N9 was detected in the ileum of one ferret at the limit of detection.

With regard to clinical changes, the D0 ferrets directly infected with H9N9 reassortant experienced a negligible increase in body temperature and a modest reduction in weight (around 2 to 3%) at 2 dpi (Fig. S6C and D) compared to Anhui/13 D0 infected ferrets which developed fever from 1 to 3 dpi and exhibited weight loss from 1 to 7 dpi, which decreased to ∼10% of their starting weight in some ferrets (Fig. S6A and B). The increase in body temperature correlated with peak viral shedding of Anhui/13 from the D0 ferrets (Fig. 9A).

The ferrets placed in contact with the directly infected Anhui/13- and reassortant H9N9-infected ferrets did not develop a significant increase in body temperature or a weight loss.

## DISCUSSION

The novel H7N9 LPAIV (Anhui/13) emerged in 2013 in China through a triple reassortment event, producing a virus with all the six internal genes derived from the G57 lineage (genotype S) of H9N2 viruses (45). Although the H7N9 virus came to prominence through its zoonotic phenotype, it is essentially an avian influenza virus (AIV) which has been circulating in avian species in China for several years (46). Cocirculation of H7N9 with several other AIV subtypes enzootic in birds in China, including H9N2, has resulted in extensive genetic reassortment that has led to the emergence of diverse H7N9 genotypes and its HPAIV variant (17, 47–52) which continue to infect humans and birds (22, 53, 54). H9 viruses have also reassorted with diverse NA subtypes, leading to nine (N1 to N9) known subtypes, including H9N9 viruses (55).

As both H7N9 and H9N2 virus circulate naturally in avian populations, it is important to determine what novel reassortants may emerge through natural coinfection with these two virus subtypes. We investigated the reassortment potential of a Chinese H7N9 virus with a G1-lineage H9N2 virus enzootic in poultry in the neighboring countries surrounding China (32, 34, 56). We have used A/Pakistan/UDL01/2008 H9N2 (G1 lineage) virus which has been previously used by our group for various chicken transmission studies (57–61) and this has helped us to decide on the dose required to establish the productive infection in chickens for our co-infection study. While UDL01 H9N2 is a decade old virus, it nevertheless remains an epidemiological representative of the G1 lineage of H9N2 viruses in countries surrounding China and the Middle East. The experimental coinfection of chickens with H7N9 Anhui/13 and a G1-lineage H9N2 UDL/08 virus resulted in the emergence of reassortant IAVs that efficiently transmitted to naive contact chickens. Many studies have investigated the generation of reassortant influenza A viruses in different hosts such as chickens (62), mallards, guinea pigs, swine (63), and embryonated chicken eggs (64), but to the best of our knowledge, this is the first study demonstrating how coinfection of chickens with H7N9 and H9N2 IAVs resulted in the emergence of a reassortant H9N9 virus with a zoonotic potential.

For two influenza viruses to reassort most efficiently in a host, the viruses must successfully establish a state of coinfection (65), for which productive infection of hosts is required (66). In view of previous studies of experimental chicken infections where a high dose was indicated for H7N9 Anhui/13 (5, 67) and a low dose was sufficient for H9N2 UDL/08 (68) to cause productive infections, we inoculated chickens with a high-virus-dose mix of Anhui/13 and UDL/08 which included predominance of the former. The observed virus shedding from directly infected chickens indicated that productive infection was established. Contact transmission indicated that fit viral progeny of unknown genotype (or genotype combinations) was produced following the mixed inoculation.

The majority of reassortant viruses derived their hemagglutinin (HA) and polymerase (PB2, PB1, and PA) genes from H9N2 UDL/08 and the neuraminidase (NA) gene from the H7N9 Anhui/13 virus. The higher polymerase activity of H9N2 in chicken cells compared to H7N9 suggests a functional advantage which would have favored the generation of reassortant H9N9 viruses containing polymerase genes from H9N2 viruses in chickens (69, 70). Although Anhui/13 H7N9 showed lower polymerase activity than UDL/08 H9N2 in chicken cells, PB2 of Anhui/13 H7N9 showed increased polymerase activity on the UDL/08 H9N2 background compared to both H7N9 and H9N2 in chicken and human cells. The Anhui/13 H7N9 originally derived its internal genes, including PB2, from poultry-adapted H9N2 viruses. The greatest polymerase activity in chicken and human cells as observed with Anhui/13 PB2 within the H9N2 background suggests that Anhui/13 PB2 may possess amino acid polymorphisms which enhance poultry and human adaptation.

The process of reassortment may either lead to attenuation of IAVs in their hosts or may selectively increase the viral fitness (71) due to “genetic tuning” of different gene segments (72). The multistep replication kinetics of reassortant viruses indicated that the M gene from Anhui/13 H7N9 (in 1+7 combination) showed increased replication in avian cells as reported earlier (73). Five reassortant H9Nx viruses, namely, genotypes 118, 119, 121, 122, and 124 were more lethal in embryonated chicken eggs compared to the progenitor H7N9 and H9N2 viruses. This increased mortality in chicken embryonated eggs could be partly due to increased replication in avian cells, which was greater for genotypes 119 and 121 and was also observed for genotypes 122 and 124 (although not significantly). The M gene from Anhui/13 (in 1+7 combination) also showed increased replication in MDCK cells compared to UDL/08, while the Anhui/13-origin M-gene in an H9N2 genetic backbone has been shown to increase virulence in a mouse infection model (74).

To select a reassorted genotype for further evaluation with respect to zoonotic potential, the assessment was done by identifying the percent genotype frequencies in the swab samples, and all the highly abundant genotypes were then compared for their replication kinetics in human A549 cells. The H9N9 (genotype 122) was chosen as a viable reassortant which successfully emerged *in vivo* from infected chickens as a relatively abundant genotype having greater embryo lethality in chicken eggs and having more dynamic replication in A549 cells, thereby guiding its selection as the likely candidate for assessing zoonotic potential.

For an AIV to cross the species barrier and infect humans requires an adaptive change which includes a shift in binding preference of viral glycoproteins toward human receptors (6SLN) (75) and an increased stability reflected in a lower pH of endosomal membrane fusion (76). The receptor binding phenotype of reassortant H9N9 virus (genotype 122) showed a stronger binding for human 6SLN and avian 3SLN sialoglycans compared to the parental H9N2 virus, but its strongest binding avidity was toward the avian-like 3SLN(6-su) receptor analogue. Compared to the H7N9 parental virus, the binding preference of the genotype 122 H9N9 virus was comparable for the 6SLN but weaker for 3SLN sialoglycans, but the reassortant H9N9 again showed its increased binding toward the 3SLN(6-su) receptor analogue. Viruses with preferable binding toward 3SLN(6-su) may have an increased propensity for circulation in terrestrial poultry (77, 78), and this observation was reflected in the successful generation and transmission of these H9N9 genotypes in chickens in our *in vivo* experiment. Previous analysis of N9 NA showed that hemadsorption sites could be responsible for increasing the overall avidity of the virus toward the sialoglycans (79, 80). Thus, increased viral replication of reassortant viruses bearing the NA (N9) of Anhui/13 origin in human A549 cells and MDCK cells may be a consequence of the N9 enhancing the binding avidity to the human-like 6SLN receptors. The H9N2 and genotype 122 H9N9 viruses were found to have an optimal pH fusion of 5.4, and different NAs did not affect the fusion pH (81). The pH fusion of H9N9 was slightly lower compared to that of Anhui/13 H7N9, having an optimal pH fusion 5.6 as seen previously (82). The results suggested that the reassortant H9N9 virus has a relatively more acid stable HA, similar avidity for human-like (6SLN) and less avidity for avian-like (3SLN) compared to Anhui/13 H7N9 virus. Further, the H9N9 virus has strongest binding toward 3SLN(6-su) receptors, thereby maintaining adaptation of such viruses for poultry (78) while also enabling additional zoonotic potential (83, 84).

Ferrets are widely recognized as an effective animal model for evaluation of IAV transmission and pathogenesis in humans (85). Thus, to investigate the possible *in vivo* consequences of variable *ex vivo* receptor binding of a reassortant H9N9 virus, genotype 122 was assessed for infection and transmission in ferrets. This reassortant H9N9 virus was compared with one parental virus, H7N9 Anhui/13, for virus shedding, dissemination in internal organs, including the upper and lower respiratory tract, and pathogenesis. Interestingly, the reassortant H9N9 virus showed similar peak titers to those of H7N9 in the directly infected (D0) ferrets, although D0 ferrets infected with H7N9 continued to shed virus for a longer duration. Ferrets infected with either H7N9 or reassortant H9N9 demonstrated that both of these viruses successfully replicated in the nasal cavity to a similar degree and caused lesions in the nasal mucosa (Fig. S4). This can be explained due to the predominance of α-2,6 sialic acid receptors in the upper respiratory tract of ferrets (86, 87) and our receptor binding results showing comparable binding in both H7N9 and H9N9 toward 6 SLN sialic acid receptor analogues.

H7N9-infected ferrets showed more lung pathology (Fig. S5) and viral RNA compared to H9N9-infected ferrets. These results corroborated our receptor binding analysis showing increased binding in H7N9 toward 3 SLN sialic acid receptor analogues compared to H9N9 and due to the predominance of α-2,3 sialic acid receptors in the lower respiratory tract of ferrets as reported previously (86, 87). The D0 and R1_DC_ ferrets in both the H7N9- and H9N9-infected groups seroconverted against the homologous viruses. Interestingly, among the R1_In_ ferrets, those in the H9N9 contact group seroconverted, while the ferrets in H7N9 contact group did not. Although H9N9 virus was not detected in the R1_In_ ferrets by RT-qPCR, the seropositive findings suggested that H9N9 may have initiated a very limited or highly localized infection following respiratory droplet exposure. Such a restricted infection may have been below the sensitivity of RT-qPCR detection but nevertheless elicited seroconversion.

Sequence analysis of the variant H9N9 viruses from the directly in-contact ferrets, performed by next-generation sequencing, revealed one nonsynonymous amino acid polymorphism at the consensus level in the HA gene. This polymorphism resulted in an amino acid change from an alanine to threonine at amino acid position 180 (A180T in mature H9 peptide numbering; A190T in mature H3 peptide numbering) (data not shown). This amino acid change is in the 190-helix proximal to the receptor binding site and has been shown to increase the binding avidity of H9N2 more than 3,500-fold and 20-fold toward avian (3SLN) and human (3SLN) receptors, respectively (88). It has therefore been suggested that this mutation may be associated with mammalian adaptation (88) and may have arisen to compensate for the stalk deletion present in the N9 glycoprotein (2) of the H9N9 reassortant in order to maintain the crucial HA-NA balance for successful viral entry and exit from cells during the infection cycle (89).

The number of human infections associated with H7N9 virus in China have been reportedly reduced after implementation of extensive poultry vaccination during autumn 2017 (90). However, vaccination has further led to emergence of vaccine escape mutants, making the control of H7N9 virus in poultry more challenging (91). Further, the H7N9 virus has been reassorting with enzootic H9N2 viruses in eastern China since 2014 (72, 92), leading to the emergence of H9N9 viruses in natural ecosystems bearing internal genes from Anhui/13 H7N9 during 2016 to 2019 (93). Due to the very low cloacal shedding observed in a number of infected contact chickens, the segment-specific RT-qPCRs were unable to successfully genotype all the viral segments from the cloacal samples (Fig. 2B). Therefore, it was not possible to identify whether any H9N9 reassortants containing the polymerase genes from Anhui/13 were generated in our study. Whether internal genes from Anhui/13 H7N9 can provide a fitness advantage, compared to the internal genes from G1 lineage H9N2 viruses, needs further investigation.

Collectively, our data show that cocirculation of H7N9 and H9N2 viruses of the G1 lineage circulating in the Indian subcontinent and the Middle East could lead to the emergence of novel reassortant H9N9 viruses which can transmit in poultry with additional zoonotic potential. Further evolutionary adaptation could enable efficient transmission to and between mammalian species such as humans. Thus, cocirculation of H7N9 and H9N2 viruses in the same enzootic regions represent a credible pandemic threat, which therefore necessitates continuing vigilance, including monitoring for the emergence of novel reassorted genotypes.

## MATERIALS AND METHODS

### Safety and ethics statement.

The United Kingdom regulations categorize the H7N9 LPAIV as a specified animal pathogens order (SAPO) 4 and Advisory Committee on Dangerous Pathogens (ACDP) hazard group 3 pathogen because it is a notifiable animal disease agent and presents a zoonotic risk (https://www.hse.gov.uk/biosafety/diseases/acdpflu.pdf). In addition, the necessary UK genetic modification guidelines were considered (https://www.hse.gov.uk/biosafety/gmo/acgm/acgmcomp/index.htm); hence, all the *in vitro*, *in ovo*, and *in vivo* experiments involving H7N9 virus and derivatives were done in licensed containment level 3 facilities at The Pirbright Institute (TPI) or Animal and Plant Health Agency (APHA). All animal studies and procedures were carried out in accord with the relevant UK and European regulations and approval by the Animal Welfare Ethical Review Board (AWERB) at the APHA, Weybridge, UK.

### Viruses used in the study.

The nucleotide sequences of the different gene segments of H7N9 (A/Anhui/1/13, abbreviated to “Anhui/13”) and H9N2 (A/chicken/Pakistan/UDL-01/08, abbreviated to “UDL/08”) viruses were retrieved from Global Initiative on Sharing All Influenza Data (GISAID) webserver (https://www.gisaid.org/), and the different viral gene segments were synthesized using GeneArt (Thermo Fisher Scientific) and subcloned into the pHW2000 vector by a standard cloning technique involving BsmBI sites (94) or restriction enzyme and ligation-independent technique (95, 96). The isolate IDs of the IAVs used in the study are listed (Table S3).

### Cell culture.

The Madin Darby canine kidney (MDCK), human embryonic kidney (HEK) 293T, chicken DF-1 and adenocarcinomic human alveolar basal epithelial cells (A549) (obtained from the Central Services Unit at TPI) were maintained in Dulbecco’s modified Eagle’s medium (DMEM) (Sigma), supplemented with 10% fetal bovine serum (FBS) (Life Science Production) and 1× penicillin-streptomycin (Gibco). Primary chicken kidney (CK) cells were prepared as previously described (97) and were maintained in Eagle’s minimum essential medium (EMEM) (Sigma) containing 7% bovine serum albumin (BSA) (Sigma), 1× penicillin-streptomycin (Gibco), and tryptose phosphate broth (Sigma). All cell lines and primary cells were maintained at 37°C and 5% CO_2_.

### Virus rescue and propagation.

The viruses were rescued by reverse genetics (RG) (98, 99), confirmed by the hemagglutination (HA) assay using standard methods, and propagated in 10-day-old specific-pathogen-free (SPF) embryonated chicken eggs at 37°C for 72 h (100). The viruses were aliquoted and stored at −80°C until required, when they were diluted appropriately in DMEM (Invitrogen) for *in vitro* infections or sterile phosphate-buffered saline (PBS) for *in ovo* and *in vivo* infections.

### Quantification of viral inocula for *in vivo* experiments.

Ten-fold serial dilutions of the H7N9 and H9N2 RG viruses were made, and 100 μl of each dilution was inoculated in a group of 6 embryonated chicken eggs. These were incubated at 37°C for 72 h. Allantoic fluid was harvested from all the inoculated eggs and tested for the presence or absence of virus by the HA test (100). Egg infectious dose 50 (EID_50_) titers were calculated by the method described by Reed and Muench (101).

### Experimental design: coinfection of chickens and transmission.

Twenty-seven SPF-derived Rhode Island red chickens (procured from the National Avian Research Facility [NARF], Roslin Institute, UK) were wing bled and swabbed (oropharyngeal and cloacal) prior to the commencement of the *in vivo* experiments. In order to exclude prior or ongoing IAV infection, the serum samples were tested with the influenza A antibody enzyme-linked immunosorbent assay (ELISA) (IDEXX), which detects antibodies to the type-common NP antigen, and the swab samples were tested by M-gene RT-qPCR (detailed below). The transmission study included nine of these chickens at 3 weeks of age, referred to here as the D0 (“donor”) chickens, which were directly infected by intranasal (i.n.) inoculation with 200 μL of mixed inoculum containing 1 × 10^5^ EID_50_ of H9N2 UDL/08 and 1 × 10^8^ EID_50_ of H7N9 Anhui/13. An equal number of age-matched chickens, referred to here as the R1 (“recipient”) chickens, were introduced at 1 day postinfection (dpi) for cohousing to serve as transmission contacts. Two other groups (*n* = 3 chickens per group) served as singly infected control groups for i.n. inoculation with 200 μL of the same doses of the individual H9N2 or H7N9 RG viruses. In order to investigate the pathogenesis (organ tropism) of the mixed viral infection, another group of six age-matched chickens were housed separately and similarly infected via the i.n. route with the H7N9/H9N2 mix and were dedicated for preplanned culling of three chickens at 2 and 4 dpi for postmortem (PM) analysis. Oropharyngeal and cloacal swabs were collected daily from all the D0 and R1 chickens in the transmission study and the singly infected controls until 14 dpi, and from the six chickens which were preplanned for culling in the pathogenesis experiment. Where relevant, reference is made to the R1 chickens at “days postcontact” (dpc), which corresponded to 1 day less than the dpi. All swabs were processed in 1 mL WHO virus transport medium (VTM) (102) and stored at −80°C until further use. The chickens were monitored twice daily for clinical signs, and all were humanely euthanized by an overdose of pentobarbitone and terminally heart bled at the end of the transmission and pathogenesis experiments.

### Avian influenza virus RT-qPCRs.

Viral RNA was extracted from the swab samples and tissue homogenates by robotic and manual methods, respectively (103). For initial screening purposes, all extracted RNA samples were tested by RT-qPCR using primers and probes specific for the M gene (104) as described previously (5). A 10-fold dilution series of RNA extracted from the titrated H7N9 and/or H9N2 (known EID_50_ titer) virus was used to plot a standard curve along with the positive threshold at a threshold cycle (*C_T_*) of 36. To characterize the subtype among the progeny viruses generated after coinfection, RT-qPCRs for H7, H9, N2, and N9 genes were performed as previously described (25, 103, 105). For the six internal gene segments, the genotype was further characterized by using gene-specific primer and probes which distinguished the origin of the genetic segment of interest, i.e., Anhui/13 or UDL/08, which were detected by FAM (6-carboxyfluorescein) or HEX (6-carboxy-2,4,4,5,7,7-hexachlorofluorescein) fluorescence, respectively. The primer and probe details are listed in Table S1.

### Plaque assay and plaque purification of viruses.

To identify viable reassortant viruses that emerged after coinfection of chickens, plaque purification of virus from the contact oropharyngeal swab samples was carried out in MDCK cells. The MDCK cells in six-well plates were infected in quadruplicate with a 10-fold serial dilution of swab sample in a 500-μL volume and incubated for 1 h at 37°C. The virus inoculum was removed, and 2 mL of overlay medium containing 2% agarose was added at 37°C and allowed to set. The plates were inverted and incubated for 4 days at 37°C or until visible plaques were formed. Discrete plaques were harvested individually into 200 μL of plain DMEM using a pipette tip. RNA was extracted from the medium plaque suspensions by robotic methods and genotyped by RT-qPCR. For the *in vitro* experiments, all the viruses were titrated as PFU/mL.

### Multistep replication kinetics of viruses.

The reassortant viruses which were shed from contact chickens after coinfection were identified by RT-qPCR and rescued *in vitro* by RG. The replication kinetics of the reassortant RG viruses was assessed in CK, MDCK, and A549 cells, as previously described (106). CK and MDCK cells were infected with 0.0002 multiplicity of infection (MOI), and A549 cells were infected with 0.05 MOI of respective viruses in infection medium (DMEM containing 1× penicillin-streptomycin and 0.3% BSA). The cell supernatant from four biological replicates was harvested 24, 48, and 72 h postinfection and titrated by plaque assay.

### Minireplicon assay.

Polymerase activity was assessed *in vitro* by plasmid-based reporter gene expression as previously described (107). Chicken DF-1 cells and human HEK-293T seeded in 24-well plates were transfected with expression plasmids for different RNP combinations prepared from both the Anhui/13 and UDL/08 progenitor viruses by using Lipofectamine 2000 (Invitrogen) according to the manufacturer’s recommendations. Then, 80 ng of PCAGGS plasmid encoding PB2 and PB1, 40 ng of PCAGGS plasmid encoding PA, and 160 ng of PCAGGS plasmid encoding NP were cotransfected with 40 ng of a *Renilla* luciferase pCAGGS expression plasmid and 80 ng of a pCk-PolI-firefly plasmid expressing negative-sense firefly luciferase flanked by a noncoding region of NS under the control of chicken-specific polymerase I promoter (108). The cells were incubated for 24 h at 37°C (for HEK-293T) or 39°C (DF-1) and lysed with 100 μL of 1× passive lysis buffer. The luciferase activity in the transfected cells was measured by using a Dual Glo luciferase assay system (Promega). The polymerase activity was calculated by normalizing firefly luciferase activity relative to the *Renilla* luciferase activity. The percent relative polymerase activity (% RPA) was calculated relative to the progenitor H7N9 or H9N2 positive controls, while negative controls excluded the PB1 plasmid during transfection.

### Receptor binding.

Virus purification and biolayer interferometry were performed as described previously (88). Briefly, the embryonated egg-propagated IAVs were ultracentrifuged at 135,200 × *g* for 2 h, purified on a continuous 30% to 60% sucrose gradient, and resuspended in PBS. The purified viruses were quantified by solid-phase indirect ELISA (see the supplemental methods) and tested in an Octet RED bio-layer interferometer (Pall ForteBio, California, USA) for receptor binding against sialoglycopolymers—α2,6-sialyllactosamine (PDB no. 6SLN), α2,3-sialyllactosamine (PDB no. 3SLN), or Neu5Ac α2,3Gal β1-4(6-HSO_3_)GlcNAc [PDB no. 3SLN(6-su)], as described previously (109). Virus association with the bound receptor analogues was measured at 20°C for 30 min. Virus-binding amplitudes were normalized to fractional saturation of the sensor surface and plotted against sugar loading. The relative dissociation constant (*K_d_*), as a measure of binding to 6SLN, 3SLN, and 3SLN(6Su), was calculated.

Since biolayer interferometry involved testing of infectious virus, due to biosafety reasons, the receptor binding of H7N9 was carried out using the 2+6 reassortant of H7N9, which included internal genes from the H1N1 A/Puerto Rico/8/34 (PR8) virus. To prevent cleavage of the receptor analogues by viral NA, 100 μM (each) oseltamivir carboxylate (Roche Products Ltd., Welwyn Garden City, UK) and zanamivir (GlaxoSmithKline, Stevenage, UK) were included in the assay mixtures.

### pH stability of viruses.

The acid stability of the selected reassortant H9N9 (genotype 122) and parental H7N9 and H9N2 viruses was determined by the ability of reassortant viruses to form syncytia in infected Vero cells exposed to different pH conditions (110). The viruses diluted 2-fold in infection medium (DMEM containing 1× penicillin streptomycin) were used to infect Vero cells in 96-well format. The highest virus dilution infecting 100% of the Vero cells was calculated by immunostaining (supplemental methods). This viral titer was used to infect Vero cells in 96-well plates for the syncytium formation assays. At 16 h postinfection, cells were treated with DMEM containing 3 μg/mL TPCK (Tosyl phenylalanyl chloromethyl ketone) trypsin for 15 min and then exposed to PBS buffers with pH values ranging from 5.2 to 6.0 (at 0.1 pH-unit increments) for 5 min. The PBS buffer was then replaced with DMEM containing 10% fetal calf serum (FCS). The cells were further incubated for 3 h at 37°C to allow for syncytium formation before being fixed with an ice-cold (–20°C) methanol and acetone (1:1) mixture for 12 min and stained with 20% Giemsa stain (Sigma-Aldrich) for 1 h at room temperature. The pH at which syncytium formation was judged to be greater than 50% corresponded to the pH of viral membrane fusion.

### Experimental design: ferret infection and transmission.

Thirty male ferrets (Mustela putorius furo) were sourced from Highgate Farms, UK, at a maximum age of 3 months old and weighing between 750 and 1,000 g. The ferrets were confirmed as serologically negative to IAV by ID Screen influenza A nucleoprotein indirect ELISA (ID Vet). Ferrets were also confirmed negative for IAV ongoing infection (shedding) by testing RNA extracted from nasal washes by the M-gene RT-qPCR (104), as described above. All ferrets were microchipped (bio-thermal chip) to monitor identification number and the body temperature. Two groups of ferrets (*n* = 3 per group; the D0 ferrets) were housed in cages in separate containment rooms and directly infected via the i.n. route with 1 × 10^7^ EID_50_ of Anhui/13 (H7N9) or H9N9 (genotype 122) (Fig. S7). At 1 dpi, direct-contact ferrets (*n* = 3, i.e., the R1DC ferrets) were introduced for cohousing in the same cage with the D0 ferrets in each room. Simultaneously, indirect-contact (R1_In_) ferrets (*n* = 3) were housed in a cage adjacent to that which housed the D0 and R1_DC_ ferrets in each room (Fig. S7). Both cages in each room were separated by a double mesh which prevented direct contact between the ferrets but allowed potential IAV aerosol transmission. Each room also contained a third cage which housed three ferrets directly infected with the two IAVs, and these were culled at 4 dpi for postmortem (PM) analysis, these six being referred to as the D0_PM_ ferrets. Tissues from the D0_pm_ ferrets were put into 1 mL of PBS (10% wt/vol) and RNA extracted for testing for influenza virus RNA using M-gene RT-qPCR. All directly infected and contact-exposed ferrets were nasal washed with 1 mL of PBS (0.5 mL/nare) and similarly tested for IAV RNA using M-gene RT-qPCR until 12 dpi. The remaining ferrets were culled and cardiac bled at 12 dpi, with seroconversion to IAV assessed by the hemagglutination inhibition (HI) test using the homologous antigens, as previously described.

### Serology.

To remove the nonspecific inhibitors for HI, chicken sera were inactivated at 56°C for 30 min, while ferret sera were incubated with 4 volumes of receptor-destroying enzyme (APHA Scientific, Weybridge, UK) for 1 h at 37°C before being inactivated at 56°C for 30 min as previously described (111). Seroconversion to the subtype-specific HA antigens was identified by HI assay (100) using four hemagglutination units of homologous viruses as antigen. ID Screen influenza A nucleoprotein indirect ELISA (ID Vet) and influenza A antibody ELISA (IDEXX) were performed according to the manufacturers’ instructions.

### Histopathology and immunohistochemistry (IHC).

Formalin-fixed tissues were processed by routine histology methods. Hematoxylin and eosin (H&E) staining and immunohistochemistry (IHC) against IAV nucleoprotein (NP) were performed on serially sectioned formalin-fixed paraffin-embedded tissues as previously described (112).

### Statistical analysis.

Student’s *t* test and one-way analysis of variance (ANOVA) followed by Tukey’s or Dunnett’s multiple-comparison test was performed using Prism version 8.00 for Windows (GraphPad Software, La Jolla, CA, USA). A *P* value of <0.05 was considered statistically significant.
