# Supplementary material for: Coinfection of Chickens with H9N2 and H7N9 Avian Influenza Viruses Leads to Emergence of Reassortant H9N9 Virus with Increased Fitness for Poultry and a Zoonotic Potential
Source: J Virol. 2022 Mar 9;96(5):e01856-21. doi: 10.1128/jvi.01856-21 (PMC8906417; doi:10.1128/jvi.01856-21)
Supplement: Supplemental file 1 — Supplemental Methods; Fig. S1 to S7; Tables S1 to S3, Key Resource Table. Download jvi.01856-21-s0001.pdf, PDF file, 1.4 MB [file jvi.01856-21-s0001.pdf]

SUPPLEMENTAL INFORMATION

Supplementary methods

Immunostaining procedure - cells

Briefly, the viruses were serially diluted (two-fold) in DMEM containing 1x penicillin streptomycin to infect the Vero cells in a 96-well-plate for 1h. The inoculum was then removed and washed once with PBS before addition of DMEM medium with 5% FCS and 1x penicillin streptomycin for 16 h. The medium was removed, and cells were fixed with ice-cold methanol and acetone (1:1) mixture for 12 minutes at room temperature. The cells were washed once with PBS and blocked with blocking buffer (0.1% PBS tween containing 5% BSA) for 1hr at room temperature. The cells were incubated with anti-nucleoprotein (NP) mouse monoclonal antibody HB-65 [H16-L10-4R5 (ATCC® HB-65™)] [diluted 1:200 in dilution buffer (0.1% PBS-tween containing 0.5% BSA)] for 1 hour at room temperature. The cells were washed 4 times with wash buffer (0.1% PBS-tween) followed by incubation with horseradish peroxidase-labelled rabbit anti-mouse immunoglobulins (Dako, Denmark) (diluted 1:200 in dilution buffer). The cells were washed four times with wash buffer and developed using liquid DAB and substrate chromogen system (Dako, USA) as mentioned by the manufacturer.

Quantification of viruses using solid-phase indirect ELISA

The purified test viruses along with a reference virus [X-31 (reassortant virus carrying HA and NA from A/Aichi/2/68 and internal genes from H1N1/PR8] were diluted in carbonate bicarbonate buffer (pH 9.6) and coated on 96 well ELISA plates (Nunc MaxiSorp™) for overnight. The coated wells were permeabilized with 0.2% triton-X for 30 min at room temperature and then washed 4 times with wash buffer before incubating with blocking buffer for 1 hr. Each well was incubated with anti-NP mouse monoclonal antibody (HB65) (1:3000 dilution in dilution buffer) for 1hr. The plate was washed 4 times with wash buffer and then incubated with horseradish peroxidase labelled anti-mouse secondary antibody (Dako) (1:2000 dilution in dilution buffer) for 1 hr followed by addition of TMB substrate reagent set (BD OptEIA™). The reaction was stopped by addition of 1N H_2SO_4 and absorbance was measured at 450 nm. The concentration of the purified viruses was calculated by comparison of the estimated NP content of the reference virus X-31 as described elsewhere (Ruigrok RW. 1998; Lin et al., 2012) and expressed as picomolar (pM).

Next generation sequencing of viruses

Complete viral genome sequencing of the nasal wash sample of H9N9 infected ferrets was carried out MiSeq System (Illumina) as explained elsewhere (Puranik et al., 2020).

Supplementary Figures

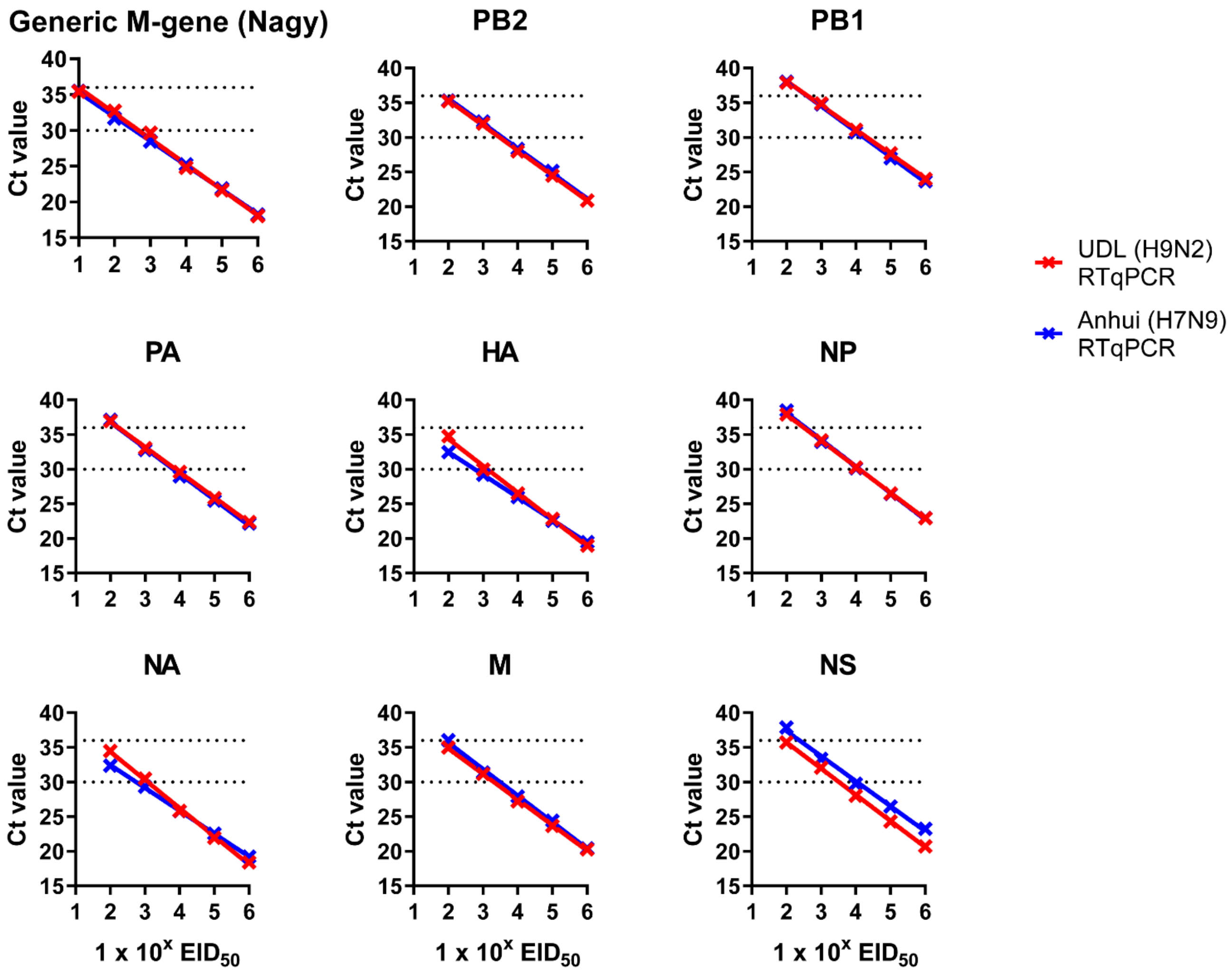

Fig. S1: Segment specific RT-qPCR standard curves. RNA was extracted from both H7N9 Anhui/13 and H9N2 UDL/08 to achieve 1x106 EID50 / reaction. A 10-fold dilution standard curve was generated for each RNA and used in the gene specific RT-qPCR assays reactions. Ct values from each assay were plotted against EID50. The primer and probes designed to specifically detect H7N9 or H9N2 gene segment had comparable efficiency.

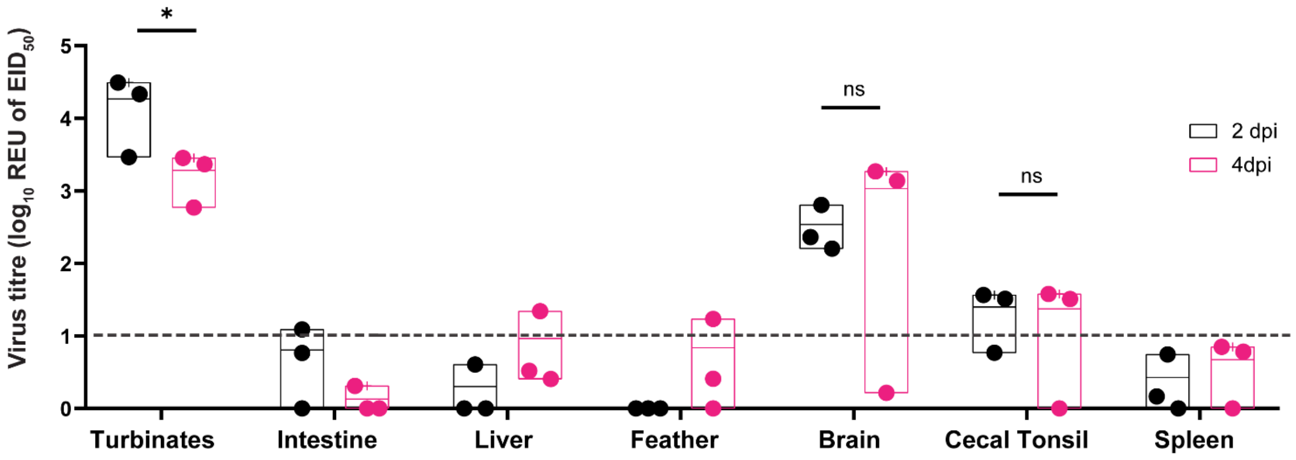

Fig. S2: Virus dissemination in internal organs of chickens directly co-infected with Anhui1/13 H7N9 and UDL01/08 H9N2. Three co-infected chickens were pre-planned for cull at (A) 2dpi and (B) 4dpi. Virus dissemination in tissues was identified using AIV-generic M-gene primers and probes (Supplementary Table S1). The Ct values were compared against an Anhui1/13 or UDL/08 RNA standards to determine relative equivalency units (REU of EID50). The dotted line represents the positive cut-off REU value.

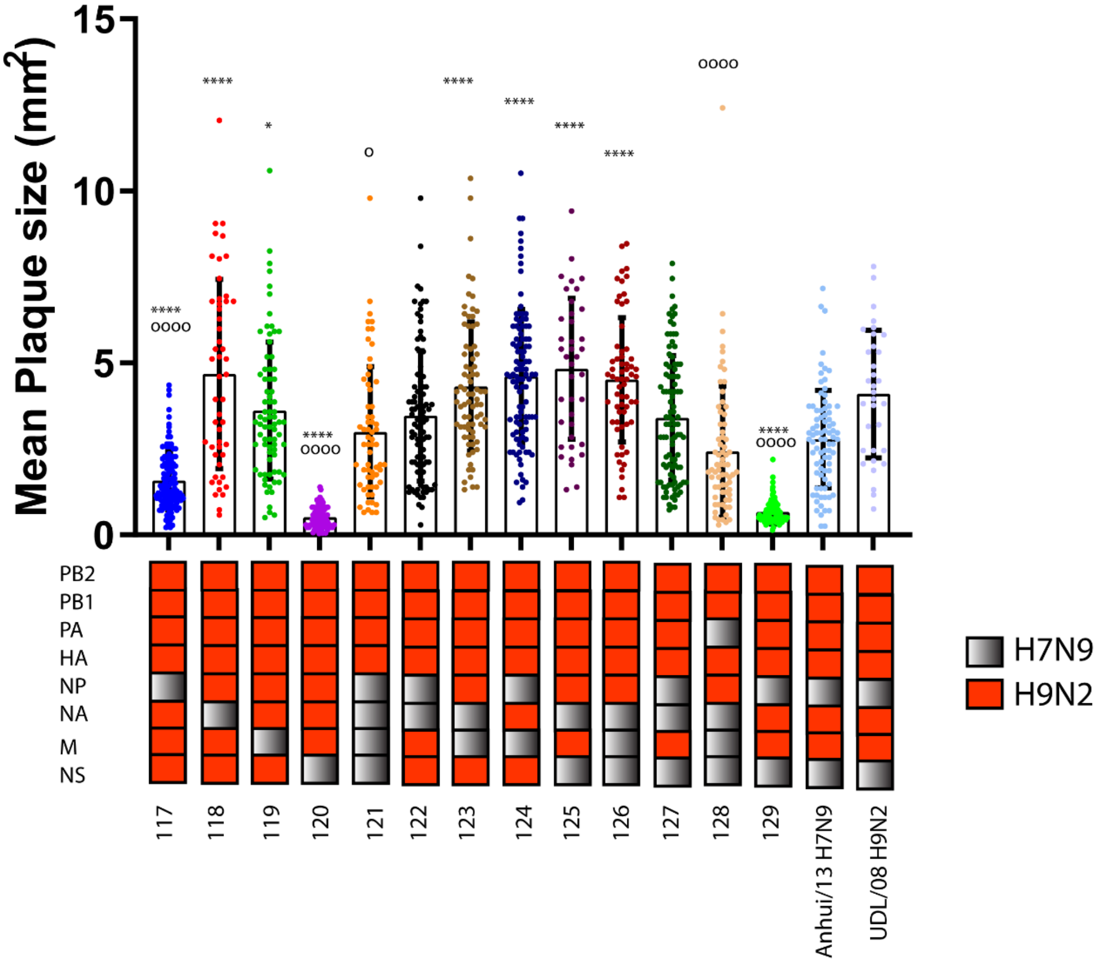

Fig. S3: Mean Plaque size (mm^2) of the reassortant H9Nx viruses on MDCK cells. Mean Plaque size (mm2) of the reassortant H9Nx viruses on MDCK cells. Plaque size was calculated in pixels using Image J software and converted to mm2. The mean plaque size of different reassortant H9Nx viruses was plotted along with Standard Error of Mean (SEM) and compared to H7N9 Anhui/13 virus and H9N2 UDL/08 virus. The mean plaque size of H9Nx viruses were compared to H7N9 virus using Dunnett's multiple comparison test (one way ANOVA). The total number of counted plaques range from 33 - 125. As compared to the H7N9 virus, the plaque size of reassortant H9N9 virus genotype 117 ($P<0.0001$), genotype 118 ($P<0.0001$), genotype 119 ($P < 0.05$), genotype 120 ($P<0.0001$), genotype 123 ($P<0.0001$), genotype 124 ($P<0.0001$), genotype 125 ($P<0.0001$), genotype 126 ($P<0.0001$) and genotype 129 ($P<0.0001$) were significantly different. While as compared to H9N2 UDL/08 virus, the plaque size of genotype 117 ($P<0.0001$), genotype 120 ($P<0.0001$), genotype 121 ($P<0.05$), genotype 128 ($P<0.0001$) and genotype 129 ($P<0.0001$) were significantly different. '**** and *' denote significance value of $P<0.0001$ and $P < 0.05$, respectively when compared to H7N9 virus. 'oooo and o' denote significance value of $P<0.0001$ and $P < 0.05$, respectively when compared to H9N2 virus.

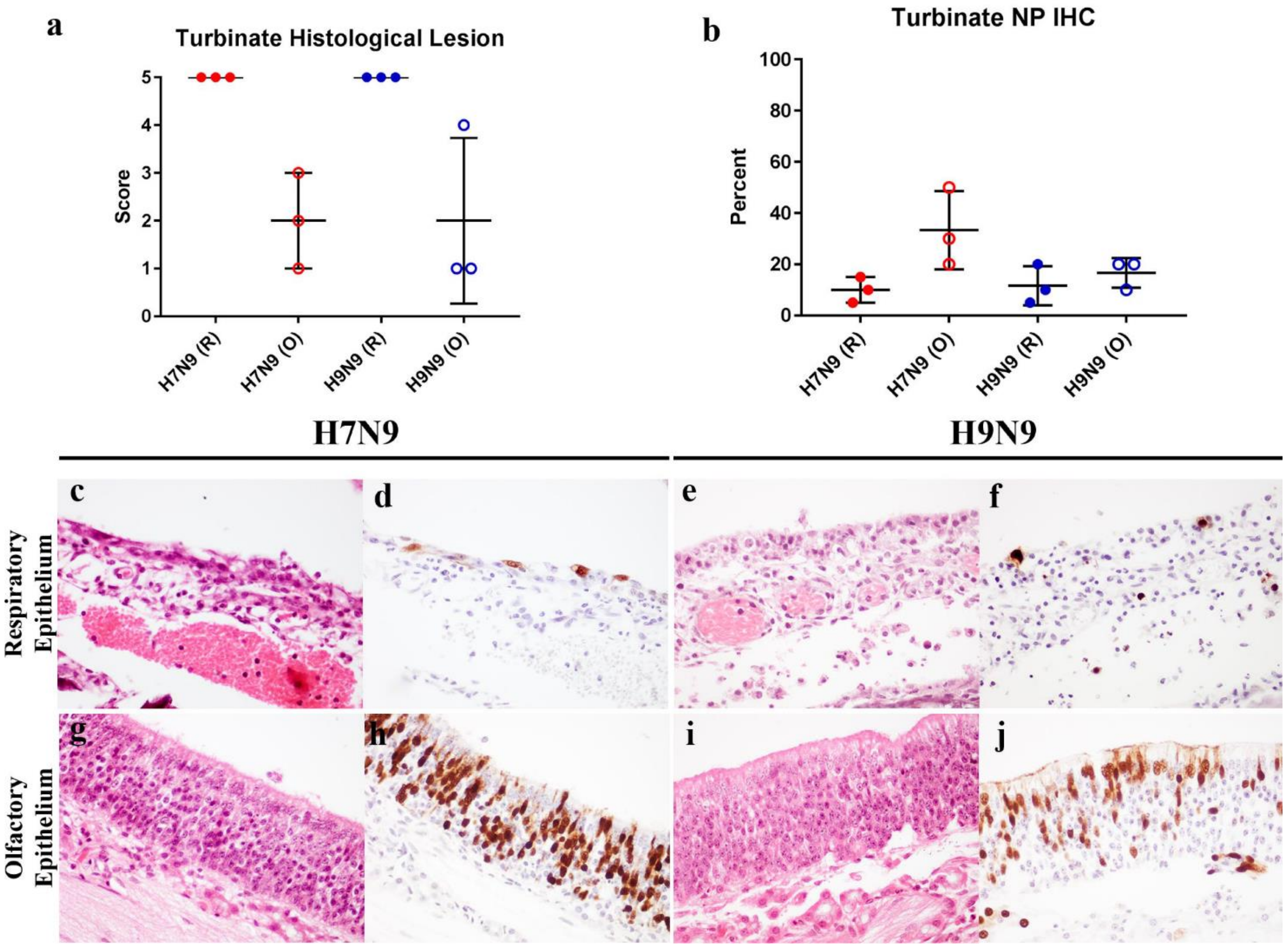

Fig. S4: Ability of H7N9 or reasortant H9N9 IAV to infect and induce pathology in the nasal turbinates of D0$_{PM}$ ferrets. Histopathological scores of nasal respiratory epithelium (R) and olfactory epithelium (O) **(a)**. Percentage of nucleoprotein (NP) immunopositive nasal epithelium (R or O) assessed by immunohistochemistry (IHC) **(b)**. Turbinate histological lesion and percentage of antigen labelling analysed with ANOVA with Tukey's multiple comparison. Representative photomicrographs of ferret respiratory (**c-f**) and olfactory epithelial mucosa (**g-j**) assessed (left to right) alternately by H&E staining and NP IHC. Images originally produced at 400x magnification.

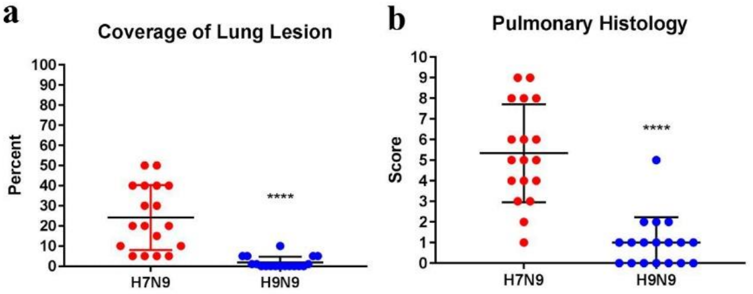

c

	H7N9			H9N9		
Ferret	263	283	316	659	673	186
Bronchial luminal epithelium	+	+	+	+	-	+
Bronchial glandular epithelium	+	+	+	+	+	+
Bronchiole epithelium	+	+	+	+	-	+
Pneumocyte	+	+	+	+	-	+
Alveolar macs	+	+	+	+	-	+

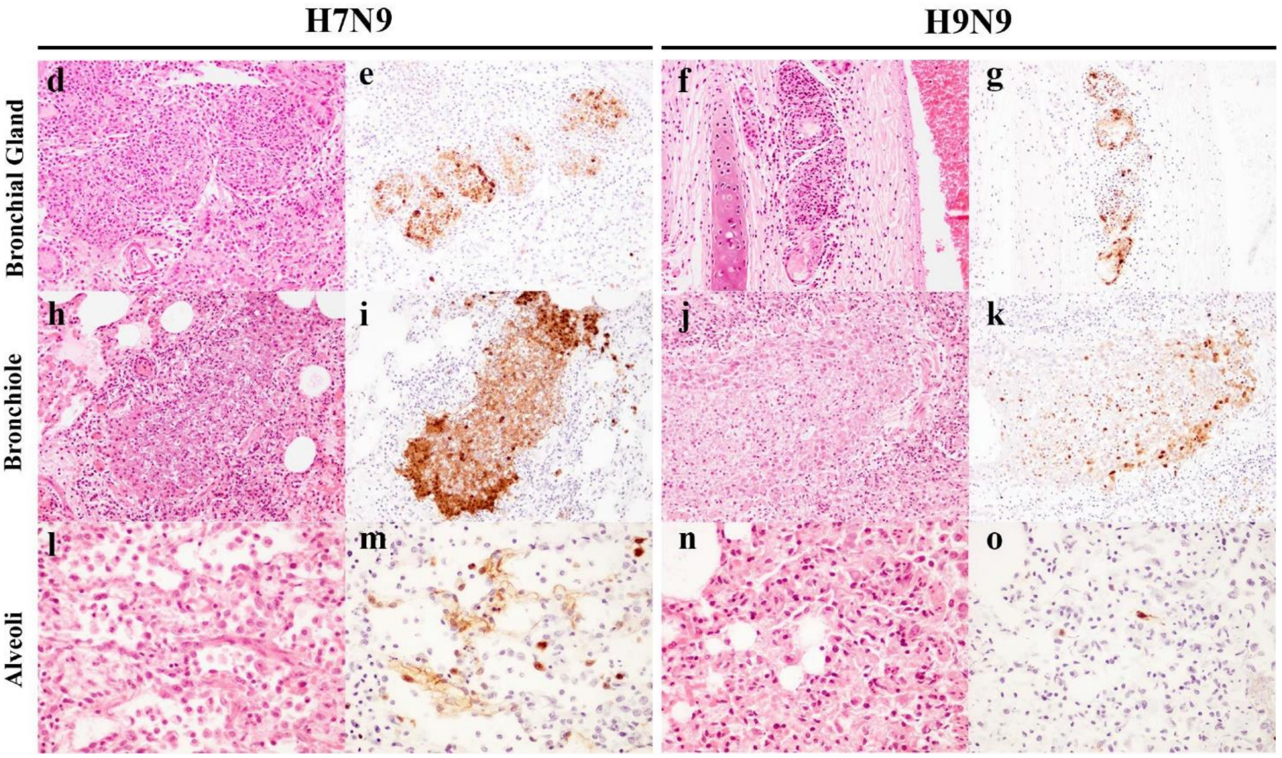

Fig. S5: Ability of H7N9 or reasortant H9N9 IAV to infect and induce pathology in the lungs of $D0_{PM}$ ferrets. Microscopy evaluation for area of lung lesions expressed in percentage **(a)** and pulmonary histopathology score **(b)**. Cellular distribution of IAV NP antigens in the lung as determined by immunohistochemistry (IHC), expressed as positive (+) or negative (-) **(c)**. Representative photomicrographs of ferret bronchial glands **(d-g)**, bronchiole **(h-k)**, and alveoli **(l-o)**. and olfactory epithelial mucosa **(g-j)** stained with H&E and NP IHC. Images taken at 200x magnification. Area of lung lesions and pulmonary histopathology score analysed with t-test. ****p<0.0001.

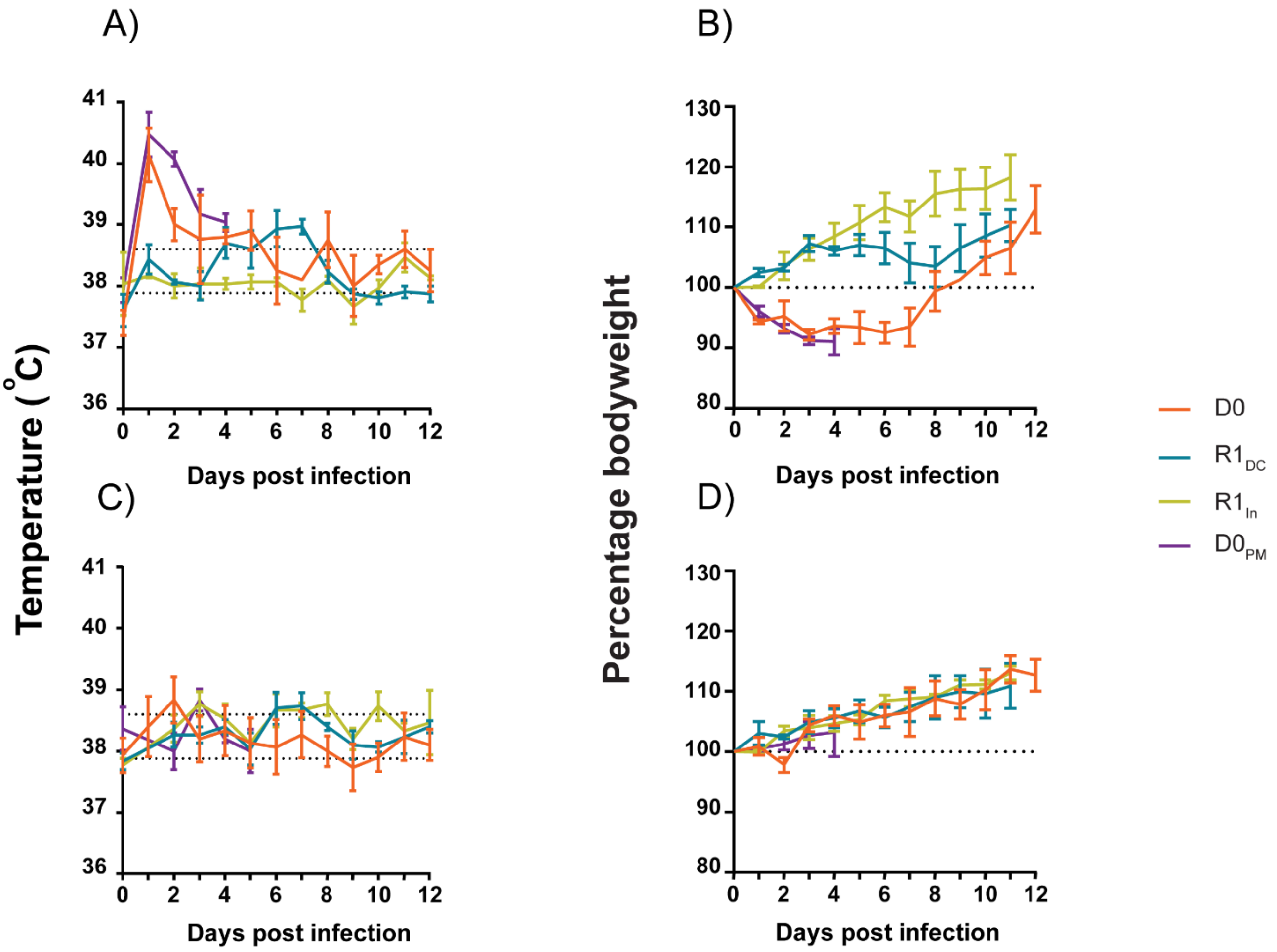

Fig S6: Changes in body temperature and body weight of ferrets infected with or exposed to Anhui/13 (A, B) and reassortant H9N9 (C, D) viruses. The infection or exposure was either through direct inoculation (D0), direct contact ($R1_{DC}$) or indirect contact ($R1_{In}$). **(A,C):** Ferrets were continuously monitored for body temperature after infection until 12 dpi / 11 dpc. The lower dotted horizontal baseline (37.8 ^{o}C) and upper dotted horizontal line (39.4 ^{o}C) indicates the normal ferret body temperature range. **(B,D):** The mean change in the body weight of the ferrets (shown as a percentage body weight) was monitored daily until 12 dpi / 11 dpc.

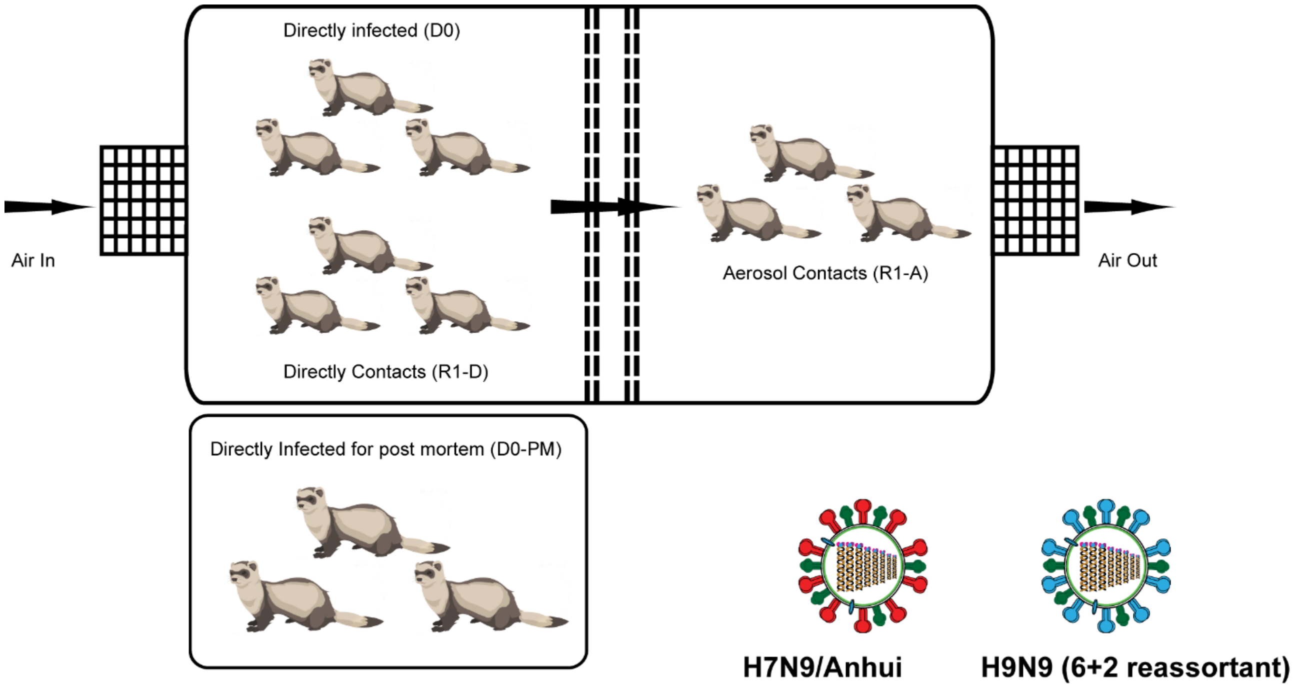

Fig S7: A schematic representation of ferret transmission experiment. Three ferrets were directly infected (D0) with H7N9 Anhui/13 virus or reassortant H9N9 virus. Contact ferrets (R1-D) were placed in contact with the directly infected ferrets in the same cage. A group of three ferrets were placed in an adjacent cage separated from the directly infected cage via a double mesh. The cages were maintained with a directional airflow from left to right (as shown by arrows above). A group of three directly infected ferrets (D0-PM) were culled on 4dpi to observe the virus dissemination in internal organs.

Supplementary Tables

Table S1: RT-qPCR primers and probes used in genotyping to identify the origins of the segments among the progeny viruses.

Target	Name	Sequence	Reference
H7	LH6H7	GGC CAG TAT TAG AAA CAA CAC CTA TGA	(Slomka et al., 2009)
	RH4H7	GCC CCG AAG CTA AAC CAA AGT AT	
	H7pro11	**FAM**-CCG CTG CTT AGT TTG ACT GGG TCA ATC T-**BHQ1**	
H9	Fwd H9	ATG GGG TTT GCT GCC	(Slomka et al., 2013)
	Rev H9	TTA TAT ACA AAT GTT GCA Y CTG	
	H9 probe	**FAM**-TTC TGG GCC ATG TCC AAT GG-**BHQ1**	
N2	IAV-N2-1367F	AGT CTG GTG GAC YTC AAA YAG	(James et al., 2019a)
	N2-1488R APHA	AAT TGC GAA AGC TTA TAT MGV CAT	
	IAV-N2-1444.1	**FAM**-CCA TCA GGC CAT GAG CCT-**BHQ1**	
N9	IAV-N9-1363F	AGY ATA GTA TCR ATG TGT TCC AG	(James et al., 2019a)
	N9-1439R	AAG TAC TCT ATT YTA GCC CCR TC	
	IAV-N9-1393FAM	**FAM**-TTC CTB GGA CAA TGG AAC TGG CC-**BHQ1**	
PB2	PB2-1F	TAT GGC CAT AAT YAA GAA ATA TAC ATC	This study
	PB2-1R	CAT CAT TTR TCT TGC TCC AAA G	
	PB2-H7N9-Pro	**FAM**- AAT CCT GCC CT+ TAG GAT GAA GT-**BHQ1**	
	PB2-H9N2-Pro	**HEX**-AAC CCT GCA CT+C AGA ATG AAA T-**BHQ1**	
PB1	PB1-1F	GCA RAT CAG AGG ATT CGT GT	This study
	PB1-1R	TCT TCT CAT TCC CTC CGA C	
	PB1-H7N9 Pro	**FAM**-T+AGA A+GC ACT AGC GAG G+AG CAT CT-**BHQ1**	
	PB1-H9N2-Pro	**HEX**-T+CGA A+AC ATT AGC GAG G+GG AAT CT-**BHQ1**	
PA	PA-1F	CAC TTA GAAGTCTGCTTCATGT	This study
	PA-1R	AWT GTT CGG TCT CTC CCT T	
	PA-H7N9 Pro	**FAM**-ACT CTG A+TT TCC ACT T+CA TCG ACG AA-**BHQ1**	
	PA-H9N2-Pro	**HEX**-ATT CGG A+CT TCC ACT T+TA TTG ATG AA-**BHQ1**	
NP	NP-1F	AGA TGT GYA CWG AAC TCA AAC T	This study
	NP-1R	TTT CAT CAA ATG CAG AGA GWA C	
	NP-H7N9 Pro	**FAM**-AGT GAC AAT GAA GGG AGG CTG ATT CAG A-**BHQ1**	
	NP-H9N2-Pro	**HEX**-AGC GAC CAA GAA GGA AGA TTG ATC CAA A-**BHQ1**	
M	M-1F	GCC ACT TGT GAG CAG ATT G	This study
	M-1R	TCC ATA GCC TTA GCC GTA GT	
	M-H7N9 Pro	**FAM**- TGA +CGC +ACA ACA TCG +GTC -**BHQ1**	
	M-H9N2-Pro	**HEX**- TGA+ TGC+ CCA ACA TCG+ TTC -**BHQ1**	
NS	NS-1F	TTG AAA GCA AAT TTC AGT GTG A	This study
	NS-1R	AGT ATG TCC TGG AAG AGA AGG	
	NS-H7N9-Pro	**FAM**-TCA ATC GGC TTG AAG CCC TGA TAC T-**BHQ1**	
	NS-H9N2-Pro	**HEX**-TTG ACC GGC TAG ACA CCT TAG TAC T-**BHQ1**	

References for the subtype-specific (HA and NA) RT-qPCRs are included, while the origins of the six internal genes are distinguished by FAM and HEX fluorescence for Anhui/13 (H7N9) and UDL/08 (H9N2), respectively. Locked nucleic acid (LNA) bases are represented with a + symbol before the base (e.g. +A)

Table S2: Distribution of virus antigens in the respiratory system of IAV infected ferret.

	**H7N9**			**H9N9**		
	**263**	**283**	**316**	**659**	**673**	**186**
Nasal Turbinate	+	+	+	+	+	+
Cervical trachea	+	+	+	-	-	-
Thoracic trachea	+	+	+	-	-	-
Left cranial lung lobe	+	+	+	+	-	+
Left caudal lung lobe	+	+	+	+	+	+
Right cranial lung lobe	+	+	+	+	-	-
Right middle lung lobe	+	+	+	+	-	+
Right caudal lung lobe	+	+	+	+	-	+
Accessory lung lobe	+	+	+	+	-	-

+ positive, - negative. Viral nucleoprotein antigen in the respiratory system of infected ferrets was detected by Immunohistochemistry (IHC) of different tissues.

Table S3: Sequence accession numbers of the progenitor AIVs used in the present study

S.No	Virus	Isolate ID
1	A/chicken/Pakistan/UDL-01/2008	EPI_ISL_29793
2	A/Anhui/1/2013	EPI_ISL_138739

The sequence of different gene segments of H9N2 UDL/08 and H7N9 Anhui/13 were retrieved from GISAID.

Key resource table

Reagent or Resource	Source	Identifier
**Antibodies**		
anti-nucleoprotein (NP) mouse monoclonal antibody	ATCC	HB-65[H16-L10-4R5 (ATCC® HB-65™)
Polyclonal rabbit anti-mouse immunoglobulin HRPO	Dako	P0260
**Bacterial or viral strains**		
A/Chicken/Pakistan/UDL-01/2008/H9N2	Iqbal Lab, TPI, UK	This study
A/Anhui/1/13/H7N9	Iqbal Lab, TPI, UK	This study
Top 10 chemically competent E. coli	ThermoFisher Scientific	C404010
**Commercial assays**		
influenza A Antibody ELISA	IDEXX	99-53101
ID Screen® Influenza A Nucleoprotein Indirect ELISA	ID Vet	FLUNPS-10P
Dual Glo luciferase assay system	Promega	E2920
**Experimental models: Cell lines**		
Madin Darby Canine Kidney (MDCK)	Central services unit, TPI, UK	Not available
human embryonic kidney (HEK) 293T	Central services unit, TPI, UK	Not available
chicken DF-1	Central services unit, TPI, UK	Not available
adenocarcinomic human alveolar basal epithelial cells (A549)	Central services unit, TPI, UK	Not available
Primary chicken kidney (CK) cells	Central services unit, TPI, UK	Not available
Vero cells	Central services unit, TPI, UK	Not available
**Experimental models: Animals/Strains**		
Chickens/Rhode Island Red	Roslin Institute, UK	Not available
Specific pathogen free eggs	Valo Biomedia, GmBH	Not available
Ferrets	Highgate Farms, UK	Not available
**Oligonucleotides and probes**		
Supplementary Table S2	Iqbal Lab, TPI, UK	This study
**Recombinant DNA**		
#875 PHW2000-PB2-UDL/08	Iqbal Lab, TPI, UK	Clements et al., 2020
#876 polInewterm_pMA-PB1-UDL/08	Iqbal Lab, TPI, UK	Clements et al., 2020
#914 pCAGGs- PB1 Pol2 Helper	Iqbal Lab, TPI, UK	Clements et al., 2020
#874 PHW2000-PA-UDL/08	Iqbal Lab, TPI, UK	Clements et al., 2020
#872 PHW2000-HA-UDL/08	Iqbal Lab, TPI, UK	Clements et al., 2020
#871 PHW2000-NP-UDL/08	Iqbal Lab, TPI, UK	Clements et al., 2020

#873 PHW2000-NA-UDL/08	Iqbal Lab, TPI, UK	Clements et al., 2020
#869 PHW2000-M-UDL/08	Iqbal Lab, TPI, UK	Clements et al., 2020
#870 PHW2000-NS-UDL/08	Iqbal Lab, TPI, UK	Clements et al., 2020
#1091 pCAGGs-PB2-UDL/08	Iqbal Lab, TPI, UK	Clements et al., 2020
#600 pCAGGs-PB1-UDL/08	Iqbal Lab, TPI, UK	Clements et al., 2020
#1093 pCAGGs-PA-UDL/08	Iqbal Lab, TPI, UK	Clements et al., 2020
#1092 pCAGGs-NP-UDL/08	Iqbal Lab, TPI, UK	Clements et al., 2020
#924 PHW2000-NS-Anhui/13	Iqbal Lab, TPI, UK	This study
#925 PHW2000-M-Anhui/13	Iqbal Lab, TPI, UK	This study
#926 PHW2000-NP-Anhui/13	Iqbal Lab, TPI, UK	This study
#927 PHW2000-PA-Anhui/13	Iqbal Lab, TPI, UK	This study
#928 PHW2000-PB2-Anhui/13	Iqbal Lab, TPI, UK	This study
#929 PHW2000-PB1-Anhui/13	Iqbal Lab, TPI, UK	This study
#1002 pCAGGs-PB2-Anhui/13	Iqbal Lab, TPI, UK	This study
#1003 pCAGGs-PB1-Anhui/13	Iqbal Lab, TPI, UK	This study
#1004 pCAGGs-PA-Anhui/13	Iqbal Lab, TPI, UK	This study
#1005 pCAGGs-NP-Anhui/13	Iqbal Lab, TPI, UK	This study
pCAGGs Renilla Luciferase	Barclay Lab, Imperial college, London	Long et al., 2016
pCk-PolI-Firefly	Barclay Lab, Imperial college, London	Long et al., 2016
**Softwares**		
Prism	GraphPad	Version 8.01
**Cell culture media and Chemicals**		
Dulbecco's Modified Eagle's medium (DMEM)	Sigma	D6429
Foetal bovine serum (FBS)	Life Science Production	S-001A-BR
Minimum Essential Medium (MEM) -10X	Sigma	M0275
Minimum Essential Medium (MEM)	Sigma	M2279
Bovine Serum Albumin (BSA)	Sigma	A7906
Penicillin-Streptomycin (10,000 U/mL)	Gibco	15140-122
Tryptose Phosphate Broth (TPB)	Sigma	T8159
Virus Transport Medium (VTM)		WHO, 2006
QIAmp viral RNA mini kit	Qiagen	52904
Low melting agarose	Oxoid	LP0028
2.5% trypsin	Gibco	15090-046
1x dissociation medium (Versene)	Gibco	15040-033
Lipofectamine 2000 reagent	Invitrogen	11668-019
Opti-MEM™ I Reduced Serum Medium	Gibco	31985-070
Sodium bicarbonate	Sigma	S8761
HEPES buffer	Sigma	H0887

Sucrose	Sigma	S0389
Neu5Aca3`(6-su)Lec-C3	Lectinity	0951-BP
3`SLN-C3	Lectinity	0036-BP
6`SLN-C3	Lectinity	0997-BP
Triton X-100	Sigma	T8787
Tween-20	Sigma	P1379
liquid DAB and substrate chromogen system	Dako	K346811-2
TMB substrate reagent set	BD OptEIA™	555214
Carbonate bicarbonate buffer	Sigma	C3041-50CAP
Receptor destroying enzyme (RDE)	APHA Scientific, UK	Not available

References:

Clements AL, Sealy JE, Peacock TP, Sadeyen JR, Hussain S, Lycett SJ, Shelton H, Digard P, Iqbal M. 2020. Contribution of Segment 3 to the Acquisition of Virulence in Contemporary H9N2 Avian Influenza Viruses. J Virol 94.

James J, Slomka MJ, Reid SM, Thomas SS, Mahmood S, Byrne AMP, Cooper J, Russell C, Mollett BC, Agyeman-Dua E, Essen S, Brown IH, Brookes SM. 2019. Development and Application of Real-Time PCR Assays for Specific Detection of Contemporary Avian Influenza Virus Subtypes N5, N6, N7, N8, and N9. Avian Dis 63:209-218

Lin YP, Xiong X, Wharton SA, Martin SR, Coombs PJ, Vachieri SG, Christodoulou E, Walker PA, Liu J, Skehel JJ, Gamblin SJ, Hay AJ, Daniels RS, McCauley JW. 2012. Evolution of the receptor binding properties of the influenza A(H3N2) hemagglutinin. Proc Natl Acad Sci U S A 109:21474-9.

Long JS, Giotis ES, Moncorge O, Frise R, Mistry B, James J, Morisson M, Iqbal M, Vignal A, Skinner MA, Barclay WS. 2016. Species difference in ANP32A underlies influenza A virus polymerase host restriction. Nature 529:101-4

Puranik A, Slomka MJ, Warren CJ, Thomas SS, Mahmood S, Byrne AMP, Ramsay AM, Skinner P, Watson S, Everett HE, Núñez A, Brown IH, Brookes SM. 2020. Transmission dynamics between infected waterfowl and terrestrial poultry: Differences between the transmission and tropism of H5N8 highly pathogenic avian influenza virus (clade 2.3.4.4a) among ducks, chickens and turkeys. Virology. 2020 541:113-123.

Ruigrok RW.1998. Structure of influenza A, B, and C viruses. Textbook of Influenza, eds Nicholson KG, Webster RG, Hay AJ (Blackwell Science, Oxford), pp 29–42.

Slomka MJ, Pavlidis T, Coward VJ, Voermans J, Koch G, Hanna A, Banks J, Brown IH. 2009. Validated RealTime reverse transcriptase PCR methods for the diagnosis and pathotyping of Eurasian H7 avian influenza viruses. Influenza Other Respir Viruses 3:151-64.

Slomka MJ, Hanna A, Mahmood S, Govil J, Krill D, Manvell RJ, Shell W, Arnold ME, Banks J, Brown IH. 2013. Phylogenetic and molecular characteristics of Eurasian H9 avian influenza viruses and their detection by two different H9-specific RealTime reverse transcriptase polymerase chain reaction tests. Vet Microbiol 162:530-42.
